# Milk intake, lactase persistence genotype, plasma proteins and risks of cardiovascular events in the Swedish general population

**DOI:** 10.1007/s10654-022-00937-7

**Published:** 2023-01-06

**Authors:** Shunming Zhang, Huiping Li, Gunnar Engström, Kaijun Niu, Lu Qi, Yan Borné, Emily Sonestedt

**Affiliations:** 1 School of Public Health, Xi’an Jiaotong University Health Science Center, Xi’an, China; 2 Nutritional Epidemiology, Department of Clinical Sciences Malmö, Lund University, Jan Waldenströms Gata 35, 21428 Malmö, Sweden; 3 Nutritional Epidemiology Institute, School of Public Health, Tianjin Medical University, Tianjin, China; 4 Cardiovascular Epidemiology, Department of Clinical Sciences Malmö, Lund University, Malmö, Sweden; 5 Department of Epidemiology, School of Public Health and Tropical Medicine, Tulane University, New Orleans, LA USA; 6 Department of Nutrition, Harvard T.H. Chan School of Public Health, Boston, MA USA

**Keywords:** Milk, rs4988235, Proteomics, Lipoprotein subfractions, Cardiovascular disease

## Abstract

To investigate the associations of milk intake (non-fermented and fermented milk), lactase persistence (*LCT*-13910 C/T) genotype (a proxy for long-term non-fermented milk intake), and gene-milk interaction with risks of cardiovascular disease (CVD) and CVD mortality. Also, to identify the CVD-related plasma proteins and lipoprotein subfractions associated with milk intake and *LCT*-13910 C/T genotype. The prospective cohort study included 20,499 participants who were followed up for a mean of 21 years. Dietary intake was assessed using a modified diet history method. Cox proportional hazards regression models were used to calculate hazard ratios (HRs) and 95% confidence intervals (CIs). After adjusting for sociodemographic and lifestyle factors, higher non-fermented milk intake was significantly associated with higher risks of coronary heart disease (CHD) and CVD mortality, whereas higher fermented milk intake was significantly associated with lower risks of CVD and CVD mortality. The genotype associated with higher milk (mainly non-fermented) intake was positively associated with CHD (CT/TT vs. CC HR = 1.27; 95% CI: 1.03, 1.55) and CVD (HR = 1.22; 95% CI: 1.05, 1.42). The association between rs4988235 genotype and CVD mortality was stronger in participants with higher milk intake than among participants with lower intake (*P* for interaction < 0.05). Furthermore, leptin, HDL, and large HDL were associated with non-fermented milk intake, while no plasma proteins or lipoprotein subfractions associated with fermented milk intake and *LCT*-13910 C/T genotype were identified. In conclusion, non-fermented milk intake was associated with higher risks of CHD and CVD mortality, as well as leptin and HDL, whereas fermented milk intake was associated with lower risks of CVD and CVD mortality.

**Supplementary Information:**

The online version contains supplementary material available at 10.1007/s10654-022-00937-7.

## Introduction

Milk is a major component in the traditional Western diet, and has been recommended in most dietary guidelines worldwide [1]. However, the health benefits of milk intake have not been well established, and concerns exist about the potential adverse health effects of high milk intake. Milk intake in Sweden is among the highest in the world [2], which provides an exceptional opportunity to investigate the association between high milk intake and human health. Due to nutritional differences between non-fermented milk and fermented milk (yogurt and cultured sour milk) [3–5], it is important to investigate them separately for health effects. In addition, studies have not shown major differences in health effects between low- and high-fat milk [2, 6].

While meta-analyses have generally not found consistent association between milk intake and mortality, several large cohorts in Sweden showed that non-fermented milk intake was associated with a higher risk of mortality [2, 7, 8]. Fermented milk intake has been associated with a lower risk of cardiovascular disease (CVD) and mortality in several studies [9–11]. In the Swedish Malmö Diet and Cancer Study (MDCS) cohort, fermented milk was associated with a lower risk of CVD during 12 years of follow-up [9]. Furthermore, the lactase persistent genetic variant *LCT*-13910 C/T (rs4988235) is highly associated with the ability to digest lactose and can be used as a proxy for the long-term milk (mainly non-fermented milk) intake [12, 13]. However, previous studies showed inconsistent results on gene-CVD analyses [14–17], and no study had investigated the gene-milk interaction on risk of CVD incidence and CVD mortality. Furthermore, the biological mechanisms underlying associations between non-fermented versus fermented milk intake and risks of CVD and mortality remain poorly understood. Proteomics provides novel insights into the biochemical pathways underlying associations of milk intake with CVD and mortality [18]. In particular, plasma proteome is a target for mechanistic study because plasma proteins are affected by environmental exposures and have important roles in biological processes including disease [19–22]. In addition, lipoprotein subfractions have been associated with the risk of CVD [23]. However, to date, no study has linked milk intake to plasma proteins using proteomics and lipoprotein subfractions. Therefore, we hypothesized that plasma proteins and lipoprotein subfractions are involved in biological processes that link milk intake to CVD events.

In the current study, we examined the associations of milk intake, *LCT*-13910 C/T genotype, and their interaction with risks of CVD incidence and CVD mortality; and identified plasma proteins and lipoprotein subfractions related to milk intake and *LCT*-13910 C/T genotype.

## Methods

### Study population

The MDCS is a population-based prospective cohort in Malmö, a city in the South of Sweden. All men and women residing in Malmö between 1991 and 1996, born between 1923 and 1950, were invited to participate in the MDCS through an advertisement or a personal letter. A total of 74,138 persons constituted the source population. Approximately 40% of the source population participated in the MDCS [24, 25]. At baseline, the participants completed a questionnaire inquiring about their socioeconomic status, dietary intake, and lifestyle factors. In addition, anthropometric measurements were taken and non-fasting blood samples were collected by trained personnel at the research site. A total of 30,446 participants completed at least one part of the baseline examination. From the MDCS cohort, a random sample of participants was invited during 1991–1994 to participate in a deeply phenotyped sub-cohort, the Malmö Diet and Cancer Cardiovascular Cohort (MDC-CC) (n = 6,103) [26]. Fasting blood samples were collected from the MDC-CC participants. All participants signed written informed consent for participation in the study. This study was approved by the Lund University Ethical Committee (approval number: LU 51/90).

**Fig. 1:**
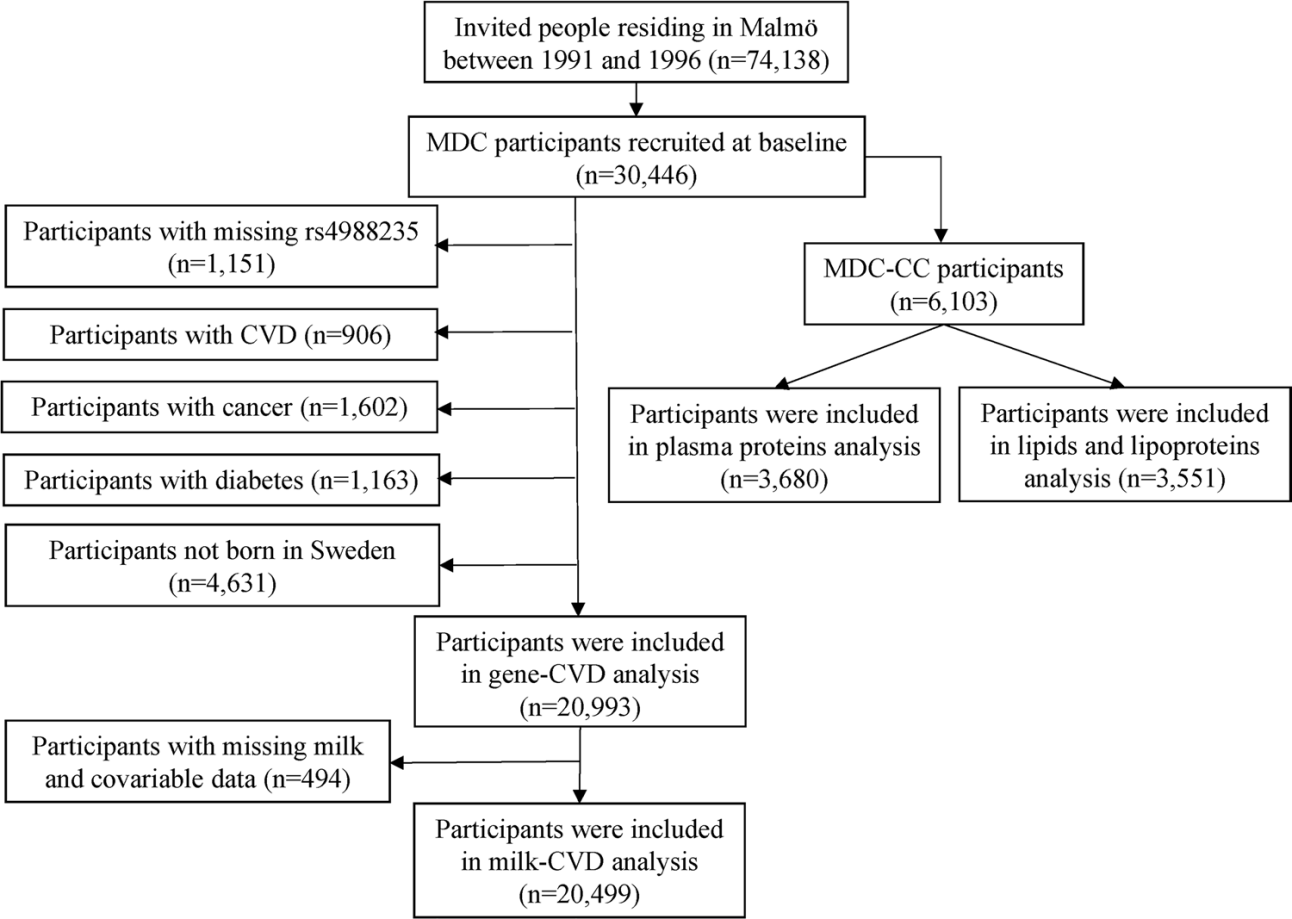
Flowchart for the study participants. CVD, cardiovascular disease; MDC, Malmö Diet and Cancer; MDC-CC, Malmö Diet and cancer cardiovascular cohort

Of the 30,446 participants in the MDCS, we excluded participants with missing rs4988235 (n = 1,151) and those with a history of CVD (coronary heart disease [CHD] or stroke, n = 906), cancer (n = 1,602), or diabetes (n = 1,163) at baseline. In addition, to reduce the risk of population stratification for the genetic analyses, those who were not born in Sweden or unknown were excluded (n = 4,631). The final analysis for the association between rs4988235 and outcomes included 20,993 participants. For milk intake and outcome analysis, we further excluded participants with missing data on milk (n = 228) and covariables (n = 266); the analytic sample included 20,499 participants. For plasma proteins and lipoprotein subfractions, the analysis was restricted to the MDC-CC participants. The available sample was 3,680 and 3,551 participants for identifying the proteins/lipoprotein subfractions associated with milk intake and rs4988235, respectively. Figure 1 shows the flowchart of the study.

### Assessment of dietary intake

At the baseline visit, habitual dietary intake was collected using a modified diet history method, which was specially developed for the MDCS [27, 28]. The method consists of three parts: a 7-day food diary, a 168-item food frequency questionnaire (FFQ), and a 45–60 min interview. The 7-day food diary recorded cooked/main meals, cold beverages, drugs, natural remedies, and dietary supplements during 7 consecutive days but did not include breakfast intake. Milk consumed as a drink and used in cooked meals was separately collected from a list of four prespecified types of milk in the 7-day food diary. The FFQ assessed foods regularly consumed and with low day-to-day variation in the past year (mainly breakfast and snacks). On the FFQ, information on milk not covered by the 7-day food diary such as milk in tea, milk in porridge, milk in cereals, milk-based spread on bread, chocolate milk, yogurt, and other fermented milk (e.g., sour milk) was collected. The interview gathered complementary information such as the type of fats used for cooking and food portion sizes in the food diary. In addition, the interview time was 60 min before August 31st 1994, and was shortened to 45 min from 1st September 1994. During the interview, the food diary and the FFQ were checked by trained interviewers according to predefined rules, so that the food diary and the FFQ did not assess overlapping foods. The average daily food (including milk) intake was estimated by combing data from the three parts. The milk was separated into non-fermented milk and fermented milk (yogurt and sour milk). Total energy intake was calculated by using the Swedish Food Database PC KOST2-93 of the Swedish National Food Administration. The validity and reproducibility of the MDCS dietary assessment method were very high [27]. The energy-adjusted correlation coefficient for total milk between the method and 18-day weighted food records (3 days every second month) was 0.83. To characterize overall diet quality, a diet index was constructed according to the Swedish Food-Based Dietary Guidelines. The diet index included five favorable components: fiber (> 2.4 g/MJ), fruit and vegetables (> 400 g/day), fish (> 300 g/week), added sugar (< 10% energy), red and processed meat (< 500 g/week). One point was given for each favorable component, and the diet index score ranged from 0 to 5. This score has been validated in our previous study and was predictive of CVD risk [29].

### Outcome ascertainment

Incident CVD was defined as the composite of CHD and total stroke. CHD, stroke, and mortality cases were ascertained using the Swedish Hospital Discharge Register, the Swedish Cause of Death Register, and the Stroke Register of Malmö [30]. CHD was defined as ICD-9 codes 410 A-410X and ICD-10 code I21 or death attributable to ischemic heart disease (ICD-9 codes: 410–414; ICD-10 codes: I20-I25). Ischemic stroke was defined as ICD-9 code 434 and ICD-10 code I63. Information on vital status and emigration was extracted from the National Tax Board. CVD mortality was defined as ICD-9 codes 390–459 and ICD-10 I00-I99. The end of the follow-up was 31st, December 2018.

### Genotyping

Genotyping was performed on the iScan System (Illumina, San Diego, CA, USA) according to the manufacturer’s recommended protocols. The quality control and imputation of rs4988235 have been described previously [7]. The *LCT* rs4988235 is a well-established genetic marker for non-fermented milk intake [7]. The rs4988235 genotype was categorized as CC (lactase deficiency), CT (lactase persistence), and TT (lactase persistence). Among 20,993 participants available for gene-outcome analysis, the minor allele frequency and the Hardy-Weinberg Equilibrium *P* value for the rs4988235 were 0.21 and 0.53, respectively.

### Plasma proteins

In MDC-CC participants, plasma proteins were measured in fasting blood samples collected at baseline and stored at -80 °C until the quantification in 2015. A total of 92 selected CVD-related proteins were measured using the Olink proximity extension assay technology (CVD-I panel, Olink Proteomics, Uppsala, Sweden). The values were presented as normalized protein expression values as arbitrary units on a log2 scale. The Olink technique had very high specificity and sensitivity [31, 32]. Four of the 92 proteins were removed before statistical analysis because a call rate was less than 75% (i.e., < 75% of the individuals had a valid measurement of that specific protein). Ultimately, 88 proteins were available for the analysis.

### Blood lipids and lipoprotein subfractions

In MDC-CC participants, fasting plasma samples were separated and stored at − 80 °C immediately after collection until analyzed. Concentrations of total cholesterol (TC), triglycerides (TG), and high-density lipoprotein cholesterol (HDL-C) were measured on a DAX 48 automatic analyzer (Bayer AB, Göteborg, Sweden) using reagents and calibrators from the supplier of the instrument. Low-density lipoprotein cholesterol (LDL-C) was calculated using the Friedewald formula [33]. These blood lipids were analyzed directly before being stored in freezer. Lipoprotein subfractions were measured later using ion mobility analysis. The ion mobility analysis method can provide accurate, reproducible, direct determination of size and concentration for a wide range of lipoprotein particles, from the small, dense HDL particles to the large, buoyant, very-low-density lipoprotein (VLDL) particles [34]. The intra- and interassay variation coefficients for LDL particles of all sizes were < 1.0%. The quality control procedure of this technique has been published elsewhere [35].

### Assessment of covariates

Information on age and sex was collected by the Swedish personal identification number. Smoking status (current, former, or never), education (the highest qualification attained), heredity score (including cancer, myocardial infarction, stroke, and diabetes; 0: no heredity or no answer in questionnaire; 1–3: heredity from father, mother and brother/sister, respectively, contributes with one “point” each), and self-reported hypertension were obtained using a structured questionnaire. Height and weight were measured by trained nurses. Body mass index (BMI) was calculated as kg/m^2^. Blood pressure was measured once from the right brachial artery using a mercury sphygmomanometer after 10 min of rest in the supine position. Hypertension was defined as systolic/diastolic blood pressure ≥ 140/90 mmHg and/or having a history of self-reported hypertension. Leisure-time physical activity was obtained by using questionnaire items adapted from the Minnesota Leisure Time Physical Activity Questionnaire. The total leisure-time physical activity volume was expressed as the weekly metabolic equivalent hours (MET-hour/week). Participants were divided into five categories: < 7.5, 7.5–15, 15–25, 25–50, and > 50 MET-hour/week. Alcohol consumption was assessed by FFQ and 7-day food diary, and then was divided into six categories: zero consumers and sex-specific quintiles of consumers. A categorical variable indicated dietary assessment version (interview time 45 or 60 min) [28]. The season of diet data collection included spring, summer, autumn, and winter.

### Statistical analysis

Baseline characteristics were presented as means ± standard deviations (SDs) or medians (interquartile ranges) for continuous variables and percentages for categorical variables. Associations of milk intake (per 100 g/day increase) with incident CVD and CVD mortality were examined using Cox proportional hazards models to calculate the hazard ratios (HRs) and 95% confidence intervals (CIs). Age was used as the timescale. Four models were fitted. Model 1 was adjusted for age, sex, dietary assessment version, season, and total energy intake. Model 2 (main model) was further adjusted for leisure-time physical activity, smoking status, alcohol consumption, education, heredity score, and diet quality index. Model 3 additionally adjusted for hypertension and BMI (potential mediators). Model 4 was adjusted for variables in model 3 plus mutually adjusted for non-fermented milk and fermented milk intake.

Age- and sex-adjusted Cox proportional hazards models were used to examine the associations of rs4988235 genotype with risks of CVD and CVD mortality. In addition, we performed a Mendelian randomization study using a two-stage least squares analysis with rs4988235 as an instrumental variable in a logistic regression model, adjusting for age and sex. Moreover, we assessed interactions on the multiplicative scale and the additive scale [36]. The multiplicative interactions between rs4988235 genotype and milk intake for risks of the outcomes were evaluated by entering their cross-products into multivariable models. Interactions on the additive scale were measured using the relative excess risk due to interaction (RERI) and 95% CI.

Linear regression models were used to identify the plasma proteins/lipoprotein subfractions associated with milk intake (non-fermented milk and fermented milk). Three progressive models were constructed. In model 1, age, sex, season, and total energy intake were adjusted. In model 2, all variables in model 1 and education, smoking status, alcohol consumption, and leisure-time physical activity were adjusted. Similar to our previous study [37], model 2 was the main model. Model 3 was further adjusted for BMI. Furthermore, to identify the plasma proteins/lipoprotein subfractions associated with the rs4988235 genotype, we used logistic regression model (CC and TC/TT) and linear regression model (CC, TC, and TT) with adjusting for age and sex. We used the Bonferroni method to account for multiple testing. *P* < 0.05/88 for plasma proteins and *P* < 0.05/16 for lipoprotein subfractions were deemed as statistically significant.

Multivariable Cox proportional hazards models were used to evaluate the associations between the identified plasma proteins/lipoprotein subfractions and incident CVD and CVD mortality. Covariates were added in a stepwise manner. Model 1 included age and sex. Model 2 included variables in model 1 plus leisure-time physical activity, smoking status, alcohol consumption, education, and heredity score. Model 3 further included BMI.

All analyses were performed using SAS software, version 9.4 (SAS Institute Inc., Cary, NC, USA). A two-sided *P* < 0.05 was set as the threshold for statistical significance.

## Results

### Baseline characteristics of participants

Table 1 presents the baseline characteristics of the study participants according to the rs4988235 genotype. The mean baseline age (SD) of the 20,499 participants was 57.8 ± 7.6 years, and 61.6% were female. Compared with the CC genotype participants, the CC/CT genotype participants consumed more non-fermented milk (median intake amounts across CC, CT, and TT genotypes: 167, 231, and 240 g/day; the coefficient of determination of simple linear regression model indicates the proportion of variability in non-fermented milk that was attributed to rs4988235 genotype is 0.0013), and they were more likely to have higher BMI and blood pressure, and slightly less likely to have a university degree.

**Table 1:** Baseline characteristics of the participants according to rs4988235 genotype (n = 20,499)

Characteristics	rs4988235 genotype		
	CC (lactase non-persistence)	CT (lactase persistence)	TT (lactase persistence)
No. of participants	966	6,872	12,661
Age (years)	57.5 ± 7.7	57.6 ± 7.6	57.9 ± 7.6
Sex (men, %)	39.4	38.0	38.5
BMI (kg/m^2^)	25.1 ± 3.7	25.4 ± 3.8	25.5 ± 3.9
SBP (mmHg)	139.4 ± 19.6	140.3 ± 19.8	140.9 ± 19.8
DBP (mmHg)	84.8 ± 9.9	85.3 ± 9.9	85.6 ± 9.9
Total energy intake (kcal/day)	2210 (1870, 2660)	2194 (1835, 2627)	2200 (1841, 2649)
Non-fermented milk (g/day)	166.5 (57.5, 317)	231.0 (88.0, 401.2)	239.7 (92.8, 407.2)
Fermented milk (g/day)	50.0 (0.0, 128.6)	50.0 (0.0, 142.9)	53.6 (0.0, 142.9)
Diet quality index	2 (1, 3)	2 (1, 3)	2 (1, 3)
LTPA (> 25 MET-hour/week, %)	53.8	53.8	52.4
Zero-consumers of alcohol (%)	5.49	5.19	5.58
University degree (%)	15.3	15.4	14.1
Smoking status (%)			
Smoker	28.2	28.2	28.1
Ex-smoker	35.4	33.6	32.8
Non-smoker	36.4	38.3	39.1
Hypertension (%)	56.9	58.9	60.6
Heredity score ^a^ (> 0, %)			
Cancer	44.1	46.8	46.6
Myocardial infarction	37.2	38.2	37.8
Stroke	25.7	26.5	26.8
Diabetes	1.97	1.82	1.94

Continuous variables are expressed as means ± standard deviations or medians (interquartile ranges) and categorical variables are expressed as percentages. BMI, body mass index; DBP, diastolic blood pressure; LTPA, leisure-time physical activity; MET, metabolic equivalent; SBP, systolic blood pressure.

^a^ 0: no heredity or no answer in questionnaire; 1–3: heredity from father, mother and brother/sister, respectively, contributes with one “point” each.

### Associations between milk intake and CVD and CVD mortality

Table 2 shows associations between milk intake and risks of CVD and CVD mortality. In the main models with adjusting for lifestyle factors (**model 2**), non-fermented milk intake was significantly associated with increased risks of CHD (HR = 1.02; 95% CI: 1.00, 1.04) and CVD mortality (HR = 1.05; 95% CI: 1.03, 1.06), but fermented milk intake was significantly associated with decreased risks of CVD (HR = 0.95; 95% CI: 0.92, 0.98) and CVD mortality (HR = 0.95; 95% CI: 0.92, 0.99). Further adjustment for hypertension and BMI as well as mutual adjustment for non-fermented milk and fermented milk (**model 4**), non-fermented milk intake was only significantly associated with increased risk of CVD mortality (HR = 1.03; 95% CI: 1.02, 1.05); fermented milk intake was only significantly associated with decreased risk of CVD (HR = 0.96; 95% CI: 0.93, 0.99).

**Table 2:** Associations between milk intake (per 100 g/day increase) and risks of CVD and CVD mortality (n = 20,499)

	Non-fermented milk	*P* value	Fermented milk	*P* value
*CHD*				
Model 1	1.04 (1.03, 1.06)	< 0.0001	0.92 (0.88, 0.96)	< 0.0001
Model 2	1.02 (1.00, 1.04)	0.04	0.97 (0.93, 1.01)	0.09
Model 3	1.01 (0.99, 1.03)	0.26	0.97 (0.93, 1.01)	0.17
Model 4	1.01 (0.99, 1.03)	0.32	0.97 (0.94, 1.01)	0.20
*Ischemic stroke*				
Model 1	1.01 (0.99, 1.03)	0.36	0.93 (0.89, 0.97)	< 0.01
Model 2	1.00 (0.98, 1.02)	0.69	0.95 (0.91, 1.00)	0.05
Model 3	0.99 (0.97, 1.01)	0.34	0.96 (0.92, 1.01)	0.09
Model 4	0.99 (0.97, 1.01)	0.26	0.96 (0.91, 1.00)	0.07
*CVD*				
Model 1	1.03 (1.02, 1.04)	< 0.0001	0.92 (0.89, 0.95)	< 0.0001
Model 2	1.01 (1.00, 1.03)	0.11	0.95 (0.92, 0.98)	< 0.01
Model 3	1.00 (0.99, 1.02)	0.52	0.96 (0.93, 0.99)	< 0.01
Model 4	1.00 (0.99, 1.02)	0.71	0.96 (0.93, 0.99)	< 0.01
*CVD mortality*				
Model 1	1.07 (1.05, 1.09)	< 0.0001	0.92 (0.88, 0.96)	< 0.0001
Model 2	1.05 (1.03, 1.06)	< 0.0001	0.95 (0.92, 0.99)	0.02
Model 3	1.04 (1.02, 1.05)	< 0.0001	0.96 (0.93, 1.00)	0.06
Model 4	1.03 (1.02, 1.05)	< 0.001	0.97 (0.93, 1.01)	0.14

Model 1: adjusted for age, sex, dietary assessment version, season, and total energy intake.

Model 2: model 1 plus leisure-time physical activity, smoking status, alcohol consumption, educational level, heredity score (including cancer, myocardial infarction, stroke, and diabetes), and diet quality index.

Model 3: model 2 plus hypertension and body mass index.

Model 4: model 3 plus mutually adjusted for non-fermented milk and fermented milk intakes.

Values are hazard ratios (95% confidence interval). CVD, cardiovascular disease; CHD, coronary heart disease.

### Associations between LCT genetic variant and CVD and CVD mortality

Table 3 displays associations between rs4988235 genotype and risks of CVD and CVD mortality. Compared with the CC genotype (lactase deficiency), CT/TT genotype (lactase persistence) was associated with a higher risk of CHD (CT/TT vs. CC: HR = 1.27; 95% CI: 1.03, 1.55) and CVD (HR = 1.22; 95% CI: 1.05, 1.42), but was not significantly associated with risks of ischemic stroke and CVD mortality. The Mendelian randomization analysis using the lactase persistence rs4988235 genotype as instrumental variable indicated that odds ratios (95% CIs) of per 100 g/day genetically predicted non-fermented milk were 1.66 (1.01, 2.72) for CHD, 1.07 (0.62, 1.85) for ischemic stroke, 1.45 (0.97, 2.16) for CVD, and 1.12 (0.77, 1.64) for CVD mortality.

**Table 3:** Associations between lactase persistent genetic variant LCT-13910 C/T (rs4988235) and risks of CVD and CVD mortality (n = 20,993)

	rs4988235 genotype			*P* for trend ^a^	rs4988235 genotype		*P* value
	CC (n = 988)	CT (n = 7,026)	TT (n = 12,979)		CC (n = 988)	CT/TT (n = 20,005)	
*CHD*							
Events	95	777	1597		95	2374	
Multivariable model ^b^	1.00 (reference)	1.19 (0.96, 1.47)	1.31 (1.06, 1.61)	< 0.01	1.00 (reference)	1.27 (1.03, 1.55)	0.02
*Ischemic stroke*							
Events	77	624	1173		77	1,797	
Multivariable model ^b^	1.00 (reference)	1.17 (0.92, 1.48)	1.17 (0.93, 1.48)	0.39	1.00 (reference)	1.17 (0.93, 1.47)	0.17
*CVD*							
Events	176	1,414	2784		176	4198	
Multivariable model ^b^	1.00 (reference)	1.18 (1.01, 1.38)	1.24 (1.07, 1.45)	< 0.01	1.00 (reference)	1.22 (1.05, 1.42)	< 0.01
*CVD mortality*							
Events	106	794	1,631		106	2,425	
Multivariable model ^b^	1.00 (reference)	1.08 (0.88, 1.32)	1.17 (0.96, 1.42)	0.02	1.00 (reference)	1.14 (0.94, 1.38)	0.20

Values are hazard ratios (95% confidence interval) unless otherwise indicated. CVD, cardiovascular disease; CHD, coronary heart disease.

^a^Test for trend based on per each increase of lactose persistence allele.

^b^Multivariable Cox proportional hazards model was adjusted for age and sex.

### Interactions between LCT genetic variant and milk on risks of CVD and CVD mortality

Table 4 presents the interactions between the *LCT* genetic variant and milk intake for risks of CVD and CVD mortality. There were significant interactions between the rs4988235 genotype and non-fermented milk intake for CVD mortality on the multiplicative and additive scale (*P* for interaction < 0.05); this positive association with non-fermented milk intake was observed only in lactase persistence (i.e., CT/TT genotype) participants. Also, the association between rs4988235 genotype and CVD mortality was stronger in participants with high intake (i.e., ≥median intake) of non-fermented milk than among participants with lower intake. And those with high non-fermented milk intake and lactase persistence had the highest risk of CVD mortality (RERI: 0.38; 95% CI: 0.09, 0.67). The interaction between the genotype and fermented milk intake for CVD and CVD mortality on the multiplicative and additive scale was also significant (*P* for interaction < 0.05). The positive association of CT/TT genotype with CVD and CVD mortality was stronger in participants with high fermented milk intake.

**Table 4:** Interactions between the lactase persistent genetic variant and milk intake on the risks of CVD and CVD mortality (n = 20,499)

	CC genotype		*P* value	CT/TT genotype		*P* value	HR (95% CI) for CT/TT within strata of milk	*P* value
	N cases/controls	HR (95% CI)		N cases/controls	HR (95% CI)			
**Non-fermented milk**								
*CHD*								
≥median intake	41/334	1.00 (reference)		1295/8579	1.21 (0.88, 1.65)	0.24	1.20 (0.88, 1.64)	0.25
<median intake	52/539	0.93 (0.61, 1.40)	0.71	1021/8638	1.16 (0.85, 1.59)	0.35	1.25 (0.94, 1.65)	0.12
HR (95% CI) for per 100 g/day milk intake within strata of genotype		0.97 (0.87, 1.07)	0.51		1.01 (0.99, 1.03)	0.35		
Measure of interaction on additive scale: RERI (95% CI): -0.03 (-0.47, 0.42); *P* = 0.45								
Measure of interaction on multiplicative scale: HR (95% CI): 0.96 (0.63, 1.46); *P* = 0.85								
*Ischemic stroke*								
≥median intake	36/339	1.00 (reference)		913/8961	0.97 (0.69, 1.35)	0.85	0.97 (0.70, 1.36)	0.87
<median intake	38/553	0.75 (0.47, 1.18)	0.22	834/8825	1.05 (0.75, 1.47)	0.78	1.41 (1.02, 1.95)	0.04
HR (95% CI) for milk intake within strata of genotype		1.01 (0.91, 1.12)	0.84		0.99 (0.97, 1.01)	0.21		
Measure of interaction on additive scale: RERI (95% CI): -0.44 (-1.08, 0.26); *P* = 0.09								
Measure of interaction on multiplicative scale: HR (95% CI): 0.69 (0.44, 1.10); *P* = 0.12								
*CVD*								
≥median intake	75/300	1.00 (reference)		2231/7643	1.14 (0.9, 1.43)	0.27	1.14 (0.90, 1.43)	0.28
<median intake	95/496	0.89 (0.65, 1.20)	0.43	1860/7799	1.14 (0.9, 1.44)	0.28	1.28 (1.05, 1.58)	0.02
HR (95% CI) for milk intake within strata of genotype		0.99 (0.92, 1.07)	0.77		1.00 (0.99, 1.02)	0.80		
Measure of interaction on additive scale: RERI (95% CI): -0.13 (-0.47, 0.22); *P* = 0.24								
Measure of interaction on multiplicative scale: HR (95% CI): 0.89 (0.65, 1.21); *P* = 0.44								
*CVD mortality*								
≥median intake	39/336	1.00 (reference)		1384/8490	1.42 (1.03, 1.95)	0.03	1.41 (1.02, 1.93)	0.04
<median intake	65/526	1.33 (0.89, 1.97)	0.17	973/8686	1.24 (0.90, 1.72)	0.18	0.93 (0.72, 1.20)	0.57
HR (95% CI) for milk intake within strata of genotype		0.92 (0.82, 1.03)	0.15		1.04 (1.02, 1.05)	< 0.0001		
Measure of interaction on additive scale: RERI (95% CI): 0.38 (0.09, 0.67); *P =* 0.01								
Measure of interaction on multiplicative scale: HR (95% CI): 1.51 (1.01, 2.27); *P* = 0.046								
**Fermented milk**								
*CHD*								
≥median intake	40/449	1.00 (reference)		1075/9054	1.34 (0.98, 1.84)	0.07	1.35 (0.98, 1.85)	0.66
<median intake	53/424	1.25 (0.83, 1.88)	0.30	1241/8163	1.43 (1.05, 1.97)	0.03	1.15 (0.87, 1.51)	0.33
HR (95% CI) for per 100 g/day milk intake within strata of genotype		0.97 (0.87, 1.07)	0.51		1.01 (0.99, 1.03)	0.29		
Measure of interaction on additive scale: RERI (95% CI): 0.12 (-0.24, 0.48); *P* = 0.74								
Measure of interaction on multiplicative scale: HR (95% CI): 1.17 (0.78, 1.77); *P* = 0.48								
*Ischemic stroke*								
≥median intake	31/458	1.00 (reference)		840/9289	1.36 (0.95, 1.95)	0.09	1.38 (0.96, 1.97)	0.08
<median intake	43/434	1.39 (0.88, 2.21)	0.16	907/8497	1.48 (1.03, 2.12)	0.03	1.04 (0.76, 1.41)	0.82
HR (95% CI) for milk intake within strata of genotype		1.02 (0.92, 1.13)	0.77		0.99 (0.97, 1.01)	0.27		
Measure of interaction on additive scale: RERI (95% CI): 0.20 (-0.17, 0.56); *P* = 0.85								
Measure of interaction on multiplicative scale: HR (95% CI): 1.29 (0.80, 2.06); *P* = 0.30								
*CVD*								
≥median intake	68/421	1.00 (reference)		1951/8178	1.47 (1.16, 1.87)	< 0.01	1.48 (1.16, 1.89)	< 0.01
<median intake	102/375	1.50 (1.11, 2.05)	0.01	2140/7264	1.58 (1.24, 2.01)	< 0.001	1.03 (0.85, 1.26)	0.74
HR (95% CI) for milk intake within strata of genotype		1.00 (0.92, 1.07)	0.89		1.00 (0.99, 1.02)	0.61		
Measure of interaction on additive scale: RERI (95% CI): 0.26 (0.04, 0.49); *P* = 0.02								
Measure of interaction on multiplicative scale: HR (95% CI): 1.41 (1.03, 1.92); *P* = 0.03								
*CVD mortality*								
≥median intake	41/448	1.00 (reference)		1139/8990	1.39 (1.02, 1.90)	0.04	1.38 (1.01, 1.89)	0.04
<median intake	63/414	1.53 (1.03, 2.28)	0.03	1218/8186	1.41 (1.03, 1.93)	0.03	0.93 (0.72, 1.20)	0.58
HR (95% CI) for milk intake within strata of genotype		0.93 (0.83, 1.04)	0.22		1.04 (1.02, 1.06)	< 0.0001		
Measure of interaction on additive scale: RERI (95% CI): 0.33 (0.06, 0.60); *P =* 0.02								
Measure of interaction on multiplicative scale: HR (95% CI): 1.52 (1.01, 2.26); *P* = 0.04								

Multivariable Cox proportional hazards model was adjusted for age, sex, dietary assessment version, season, total energy intake, physical activity, smoking status, alcohol consumption, education, heredity score (including cancer, myocardial infarction, stroke, and diabetes), diet quality index, hypertension, and body mass index, and each other for non-fermented milk and fermented milk intakes.

HR, hazard ratio; CI, confidence interval; CVD, cardiovascular disease; CHD, coronary heart disease; RERI, relative excess risk due to interaction.

### Associations of milk intake with plasma proteins and lipoprotein subfractions

**Fig. 2:**
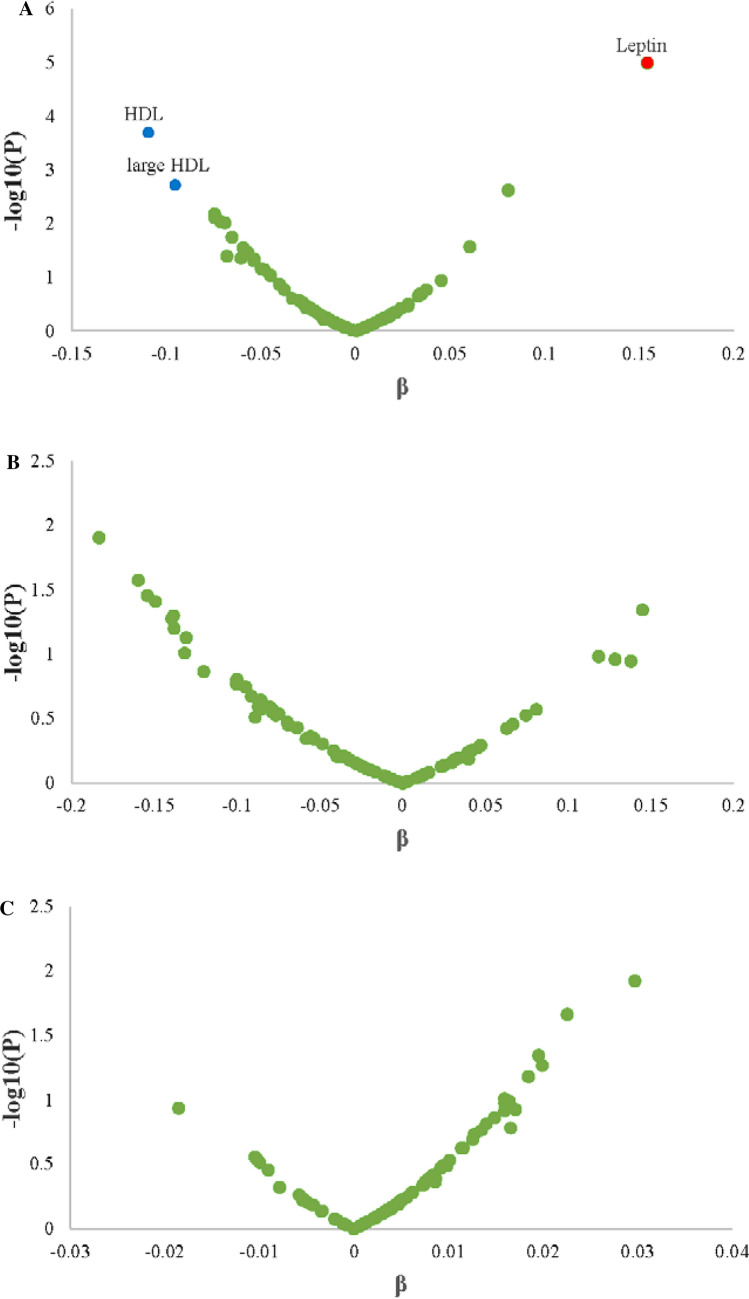
Volcano plots of the relationships between non-fermented milk intake (**a**), fermented milk intake (**b**), and *LCT*-13910 C/T genotype (**c**) and plasma proteins/lipoprotein subfractions. For A and B, multiple linear regression models adjusted for age, sex, season, total energy intake, education, smoking status, alcohol consumption, and leisure-time physical activity. For C, multiple linear regression models adjusted for age and sex. Red dots indicate the plasma proteins positively and statistically significantly associated with non-fermented milk (Bonferroni corrected *P* < 0.05/88), and blue dots indicate lipoprotein subfractions inversely and statistically significantly associated with non-fermented milk (Bonferroni corrected *P* < 0.05/16). Green dots represent plasma proteins/lipoprotein subfractions not statistically associated with non-fermented milk, fermented milk, or *LCT*-13910 C/T genotype

After adjusting for age, sex, season, and total energy intake and controlling for multiple testing (**model 1**), leptin was found to be positively associated with non-fermented milk intake, while HDL and large HDL were inversely associated with non-fermented milk intake. Further adjustment for education, smoking status, alcohol consumption, and leisure-time physical activity did not appreciably change the results. However, additional adjustment for BMI made such associations attenuate. Supplementary Table 1 and Fig. 2a show the plasma proteins and lipoprotein subfractions associated with non-fermented milk intake, adjusting for lifestyle factors.

In model 1, fermented milk intake was inversely associated with interleukin-18 (IL18), urokinase plasminogen activator surface receptor (UPAR), matrix metalloproteinase-12 (MMP12), medium and large VLDL. Further adjustment for lifestyle factors made such associations become not statistically significant. Supplementary Table 2 and Fig. 2b show the associations of plasma proteins and lipoprotein subfractions with fermented milk intake.

### Associations of LCT genetic variant with plasma proteins and lipoprotein subfractions

There were no statistically significant associations found with the rs4988235 genotype and plasma proteins/lipoprotein subfractions either in logistic regression models or in linear regression models (Supplementary Table 3 and Fig. 2c).

### Associations of the identified plasma proteins and lipoprotein subfractions with outcomes

Supplementary Table 4 shows the associations between the identified plasma proteins and lipoprotein subfractions in relation to non-fermented milk intake and outcomes. After adjusting for age and sex, leptin was positively associated, but HDL and large HDL were inversely associated with risks of CHD, CVD, and CVD mortality. Further adjustment for lifestyle factors yielded similar results. However, such associations were attenuated more markedly by additional adjustment for BMI.

## Discussion

In this population-based prospective cohort study with a mean follow-up time of > 20 years, we observed that non-fermented milk intake was positively associated with risks of CHD and CVD mortality, whereas fermented milk intake was inversely associated with risks of CVD and CVD mortality. In addition, the *LCT* rs4988235 genotype, a proxy for long-term non-fermented milk intake, was positively associated with risks of CHD and CVD. There was an interaction between the genotype and milk for CVD mortality. In addition, leptin and large HDL were associated with non-fermented milk.

An initial report published in 2011 for this cohort found that fermented milk intake was significantly associated with a lower risk of CVD, but non-fermented milk intake was not [9]. With an additional decade of follow-up, we showed that higher non-fermented milk intake was significantly associated with a higher risk of CHD and CVD mortality, but fermented milk intake was significantly associated with lower risks of CVD and CVD mortality. Other studies from Finland and Sweden also reported that non-fermented milk intake had positive associations with CHD [38] and CVD mortality [8]. In contrast, studies from low-income and middle-income countries showed an inverse association between milk intake and cardiovascular events [10]. For example, studies in East Asia (most individuals were lactase non-persistence) with low milk intake indicated that non-fermented milk intake was inversely associated with CVD mortality [39, 40]. This difference between North Europe and low-middle countries might be partly due to higher milk consumption in North Europe (non-fermented milk mean: 278 g/day in the MDCS) compared with low-middle countries (milk mean: <100 g/day) [10]. Furthermore, in line with previous studies [41], the current study indicated inverse associations of fermented milk intake with incident CVD and CVD mortality. These findings further highlight that non-fermented milk and fermented milk may have distinct associations with the risk of CVD events.

Several potential mechanisms have been proposed to explain the observed positive associations between non-fermented milk intake and risks of CHD and CVD mortality. First, the main carbohydrate in non-fermented milk is lactose [1]. Lactose can be digested into D-glucose and D-galactose. Animal experiments have shown that D-galactose could increase oxidative stress and chronic low-grade inflammation levels [42–44], which might result in adverse health outcomes. Moreover, we aimed to among 88 plasma proteins (including inflammatory markers and other disease-associated markers) and 16 lipoprotein subfractions identify markers associated with milk intake to provide clues to underlying biological pathways, and found that non-fermented milk intake was positively associated with plasma leptin levels. Leptin has been linked to endothelial dysfunction, activation of the sympathetic nervous system, and deleterious effects on the underlying vasculature [45]. Moreover, prospective studies have associated higher baseline leptin levels with the risk of CHD and CVD [46–48], consistent with our study and supporting our findings. Third, milk intake contains saturated fatty acids, which can increase blood lipid levels. However, our study indicated that only non-fermented milk intake was inversely associated with HDL and large HDL cholesterol levels.

Contrary to non-fermented milk, fermented milk intake was inversely associated with risks of CVD and CVD mortality. A possible explanation is that fermentation reduces the galactose content in milk [49], while galactose is harmful [50]. In addition, fermented milk is rich in bacteria with probiotic properties [5]. These bacteria may have anti-inflammatory, antioxidant, and immunomodulatory activities after absorbing into the gut. Moreover, the current study shows that fermented milk intake was inversely associated with IL18, UPAR, and MMP12 in the basic adjusted model, all of which have been found to be inflammatory biomarkers [51–53].

In gene-outcome analyses, we observed significant associations between *LCT* rs4988235 (a genetic marker of non-fermented milk) and risks of CHD and CVD. However, a study conducted in a Danish population of 98,529 participants found that the T allele of rs4988235 was not associated with the risk of ischaemic heart disease and myocardial infarction [14]. Likewise, another European descent study showed that there was no association of rs4988235 genotype with the risk of CHD (OR = 0.99; 95% CI: 0.95, 1.03) and total stroke (OR = 1.02; 95% CI: 0.99, 1.05) [17]. In contrast, a meta-analysis of the data from three large-scale population-based studies (1958 British Birth Cohort, Health and Retirement Study, and UK Biobank) indicated that the rs4988235 genotype T allele was associated with a lower risk of coronary artery disease (OR = 0.86, 95% CI: 0.75, 0.99) [16]. The inconsistent associations of rs4988235 with CHD and CVD may be partly due to big differences between populations with regard to milk intake. Indeed, when conducting analyses with the genetic variant in different strata of milk intake, the association between the genotype and CVD mortality was stronger in participants with high non-fermented milk intake than among participants with lower intake. Furthermore, a Danish study showed no association between rs4988235 genotype and CVD mortality [15], similar to our study. The Mendelian randomization analysis in our study indicated that genetically predicted non-fermented milk was significantly associated with increased risk of CHD, but not ischemic stroke, CVD, and CVD mortality. Previous Mendelian randomization studies did not observe significant association between milk intake and CHD, and stroke, CVD, and CVD mortality [14, 15, 17]. The discrepancy may be due to the previous studies did not take into account the type of milk consumed.

Interestingly, we observed that the rs4988235 genotype significantly modified the association of milk intake with CVD mortality. Among participants bearing CT/TT genotype, milk intake was associated with an increased risk of CVD mortality; but among CC genotype participants, an inverse association was observed, albeit without statistical significance due to the small sample size. A potential explanation for high milk intake is protective (at least almost significant) only among CC genotype (those that cannot break down lactose) is that undigested lactose can pass through the small intestine into the colon and behave as a fiber among lactase non-persistence individuals [54, 55]. And fiber has beneficial health effects [56]. Another explanation was that CC genotype participants (lactase non-persistence) might consume less milk.

Unlike results for non-fermented milk, we did not identify any plasma proteins and lipoprotein subfractions associated with the rs4988235 genotype. The genetic marker can be used as a proxy for long-term intake of lactose-containing foods (i.e., mainly non-fermented milk) which is less influenced by confounding compared with self-reported intakes. Given that the rs4988235 genotype is not a perfect marker, this was not surprising. Indeed, the proportion of milk intake variation explained by rs4988235 is less than 1%. Further, another possible explanation was that we did not have sufficient statistical power in these analyses since only approximately 5% of the participants are CC genotype carriers. In addition, because milk drinking is the norm in Sweden, this culture may have a strong influence on milk intake among Swedish individuals, even among lactase-nonpersistent individuals.

Strengths of this study include the long duration of follow-up, large sample size comprised exclusively of Swedish adults, and the outcomes from high-quality national and local registers. More important, to the best of our knowledge, this was the first study on plasma proteins and lipoprotein subfractions implicated in CVD of habitual milk consumption and the rs4988235 genotype, a proxy for long-term non-fermented milk intake.

Nevertheless, several limitations must be acknowledged. First, because dietary intake was assessed by questionnaires and food records, measurement of milk intake would have some degree misclassification. However, such misclassification error was most likely nondifferential, attenuating the association towards the null. In addition, dietary data were collected once at baseline, and changes in dietary intake during follow-up could introduce exposure misclassification. Second, the ethnic homogeneity of the study may limit the generalizability of the findings to other nationalities and races. Third, fermented milk intake has been associated with a healthy diet and lifestyle [57, 58]. Although we adjusted for diet quality and a wide range of lifestyle factors, residual confounding was unavoidable in the current study. For example, the found associations for milk intake and CVD were very small and may well be fully explained by unmeasured or residual confounding or misclassification of exposure. Finally, the lack of an external validation cohort of the plasma proteins/lipoprotein subfractions in relation to milk intake is a potential concern. Furthermore, ion mobility lipoprotein analysis made many assumptions in calculating particle concentrations from the particle numbers “counted” by the detector [59]. Thus, some assumptions may not be valid, which could have led to the measurement error [59].

## Conclusion

In conclusion, non-fermented milk intake was positively associated with risks of CHD and CVD mortality, but fermented milk intake was inversely associated with risks of CVD and CVD mortality. The *LCT* rs4988235 genotype was positively associated with risks of CHD and CVD, and showed significant interaction with milk intake on CVD risk. We identified leptin and HDL associated with non-fermented milk. Further studies using proteomics and lipidomics might provide clues to investigate mechanisms underlying the associations between milk intake and risks of CVD and mortality.

## Electronic supplementary material

Below is the link to the electronic supplementary material.

Supplementary Material 1

## Data Availability

Supporting data are available from the corresponding author upon reasonable request but access to data must be granted by the ANDIS and MDC steering committees.
