# Supplementary material for: Milk intake, lactase persistence genotype, plasma proteins and risks of cardiovascular events in the Swedish general population
Source: Eur J Epidemiol. 2023 Jan 6;38(2):211–24. doi: 10.1007/s10654-022-00937-7 (PMC9905175; doi:10.1007/s10654-022-00937-7)
Supplement: Supplementary file 1 — Supplementary Material 1 [file 10654_2022_937_MOESM1_ESM.docx]

| **Supplementary Table 1.** Plasma proteins, lipids, and lipoprotein subfractions associated with non-fermented milk intake ^a^ | | | | | | | | | | | | |  |
| --- | --- | --- | --- | --- | --- | --- | --- | --- | --- | --- | --- | --- | --- |
|  | **Model 1** | | | | **Model 2** | | | | **Model 3** | | | |  |
|  | *β* | 95% CI | | *P* value | *β* | 95% CI | | *P* value | *β* | 95% CI | | *P* value |  |
| **Plasma proteins** |  |  |  |  |  |  |  |  |  |  |  |  |  |
| IL8 | -0.0325 | -0.0861 | 0.0212 | 0.24 | -0.0373 | -0.0906 | 0.0160 | 0.17 | -0.0393 | -0.0925 | 0.0138 | 0.15 |  |
| VEGFA | 0.0164 | -0.0371 | 0.0698 | 0.55 | 0.0091 | -0.0439 | 0.0621 | 0.74 | -0.0084 | -0.0617 | 0.0449 | 0.76 |  |
| AM | 0.0062 | -0.0486 | 0.0610 | 0.83 | 0.0005 | -0.0538 | 0.0548 | 0.99 | -0.0376 | -0.0937 | 0.0184 | 0.19 |  |
| CD40L | -0.0066 | -0.0595 | 0.0463 | 0.81 | -0.0046 | -0.0568 | 0.0476 | 0.86 | -0.0090 | -0.0611 | 0.0431 | 0.73 |  |
| GDF15 | 0.0235 | -0.0332 | 0.0802 | 0.42 | -0.0010 | -0.0596 | 0.0576 | 0.97 | -0.0103 | -0.0688 | 0.0483 | 0.73 |  |
| PlGF | 0.0180 | -0.0373 | 0.0734 | 0.52 | 0.0011 | -0.0536 | 0.0558 | 0.97 | -0.0122 | -0.0669 | 0.0426 | 0.66 |  |
| SELE | -0.0181 | -0.0722 | 0.0360 | 0.51 | -0.0192 | -0.0726 | 0.0341 | 0.48 | -0.0465 | -0.1006 | 0.0077 | 0.09 |  |
| EGF | -0.0110 | -0.0639 | 0.0419 | 0.68 | -0.0091 | -0.0612 | 0.0431 | 0.73 | -0.0131 | -0.0651 | 0.0389 | 0.62 |  |
| OPG | 0.0186 | -0.0362 | 0.0734 | 0.51 | -0.0009 | -0.0550 | 0.0533 | 0.98 | -0.0031 | -0.0571 | 0.0509 | 0.91 |  |
| SRC | -0.0194 | -0.0728 | 0.0340 | 0.48 | -0.0166 | -0.0692 | 0.0359 | 0.54 | -0.0148 | -0.0672 | 0.0375 | 0.58 |  |
| IL1ra | 0.0675 | 0.0109 | 0.1241 | 0.02 | 0.0454 | -0.0109 | 0.1017 | 0.11 | 0.0068 | -0.0528 | 0.0664 | 0.82 |  |
| IL6 | 0.0315 | -0.0222 | 0.0851 | 0.25 | 0.0210 | -0.0329 | 0.0750 | 0.44 | -0.0020 | -0.0566 | 0.0526 | 0.94 |  |
| CSTB | 0.0348 | -0.0193 | 0.0889 | 0.21 | 0.0165 | -0.0374 | 0.0704 | 0.55 | -0.0074 | -0.0620 | 0.0472 | 0.79 |  |
| MCP1 | 0.0087 | -0.0451 | 0.0625 | 0.75 | -0.0016 | -0.0547 | 0.0514 | 0.95 | -0.0115 | -0.0645 | 0.0416 | 0.67 |  |
| KLK6 | -0.0218 | -0.0752 | 0.0316 | 0.42 | -0.0401 | -0.0928 | 0.0126 | 0.14 | -0.0289 | -0.0816 | 0.0239 | 0.28 |  |
| Gal3 | 0.0416 | -0.0126 | 0.0957 | 0.13 | 0.0241 | -0.0293 | 0.0775 | 0.38 | 0.0141 | -0.0393 | 0.0675 | 0.60 |  |
| PAR1 | -0.0470 | -0.1002 | 0.0062 | 0.08 | -0.0535 | -0.1058 | -0.0012 | 0.05 | -0.0569 | -0.1090 | -0.0047 | 0.03 |  |
| TRAIL | -0.0012 | -0.0545 | 0.0521 | 0.96 | -0.0147 | -0.0673 | 0.0380 | 0.59 | -0.0312 | -0.0841 | 0.0217 | 0.25 |  |
| hK11 | -0.0532 | -0.1067 | 0.0004 | 0.05 | -0.0568 | -0.1095 | -0.0042 | 0.03 | -0.0507 | -0.1033 | 0.0019 | 0.06 |  |
| TIE2 | -0.0189 | -0.0724 | 0.0345 | 0.49 | -0.0297 | -0.0823 | 0.0229 | 0.27 | -0.0396 | -0.0922 | 0.0130 | 0.14 |  |
| TF | -0.0431 | -0.0971 | 0.0109 | 0.12 | -0.0532 | -0.1064 | -0.0001 | 0.05 | -0.0493 | -0.1023 | 0.0037 | 0.07 |  |
| TNFR1 | 0.0445 | -0.0101 | 0.0991 | 0.11 | 0.0155 | -0.0388 | 0.0697 | 0.58 | -0.0074 | -0.0623 | 0.0474 | 0.79 |  |
| PDGFsubunitB | 0.0143 | -0.0388 | 0.0673 | 0.60 | 0.0151 | -0.0373 | 0.0674 | 0.57 | 0.0093 | -0.0430 | 0.0615 | 0.73 |  |
| IL27A | 0.0137 | -0.0397 | 0.0670 | 0.62 | -0.0057 | -0.0586 | 0.0473 | 0.83 | -0.0025 | -0.0553 | 0.0504 | 0.93 |  |
| CSF1 | 0.0340 | -0.0194 | 0.0874 | 0.21 | 0.0070 | -0.0459 | 0.0599 | 0.80 | -0.0115 | -0.0647 | 0.0418 | 0.67 |  |
| CXCL1 | -0.0691 | -0.1220 | -0.0162 | 0.01 | -0.0690 | -0.1212 | -0.0168 | 0.01 | -0.0719 | -0.1240 | -0.0199 | 0.01 |  |
| LOX1 | 0.0590 | 0.0058 | 0.1121 | 0.03 | 0.0375 | -0.0161 | 0.0911 | 0.17 | 0.0336 | -0.0198 | 0.0871 | 0.22 |  |
| TRAILR2 | 0.0185 | -0.0357 | 0.0727 | 0.50 | -0.0095 | -0.0644 | 0.0455 | 0.74 | -0.0243 | -0.0794 | 0.0308 | 0.39 |  |
| FGF23 | 0.0902 | 0.0373 | 0.1430 | 0.00 | 0.0809 | 0.0288 | 0.1330 | 0.002 | 0.0659 | 0.0136 | 0.1183 | 0.01 |  |
| SCF | -0.0408 | -0.0940 | 0.0125 | 0.13 | -0.0744 | -0.1281 | -0.0207 | 0.01 | -0.0613 | -0.1152 | -0.0074 | 0.03 |  |
| IL18 | -0.0012 | -0.0557 | 0.0534 | 0.97 | -0.0243 | -0.0784 | 0.0297 | 0.38 | -0.0403 | -0.0945 | 0.0139 | 0.15 |  |
| IL6RA | 0.0210 | -0.0321 | 0.0740 | 0.44 | 0.0129 | -0.0394 | 0.0652 | 0.63 | 0.0029 | -0.0493 | 0.0552 | 0.91 |  |
| TNFR2 | 0.0681 | 0.0141 | 0.1221 | 0.01 | 0.0333 | -0.0205 | 0.0870 | 0.22 | 0.0186 | -0.0353 | 0.0725 | 0.50 |  |
| MMP3 | -0.0678 | -0.1336 | -0.0019 | 0.04 | -0.0678 | -0.1327 | -0.0030 | 0.04 | -0.0660 | -0.1306 | -0.0014 | 0.05 |  |
| HSP27 | -0.0242 | -0.0773 | 0.0288 | 0.37 | -0.0213 | -0.0735 | 0.0309 | 0.42 | -0.0271 | -0.0791 | 0.0250 | 0.31 |  |
| TNFSF14 | 0.0142 | -0.0388 | 0.0672 | 0.60 | 0.0043 | -0.0482 | 0.0568 | 0.87 | -0.0100 | -0.0626 | 0.0426 | 0.71 |  |
| PRL | -0.0032 | -0.0571 | 0.0508 | 0.91 | 0.0079 | -0.0455 | 0.0612 | 0.77 | 0.0111 | -0.0421 | 0.0643 | 0.68 |  |
| MPO | 0.0098 | -0.0434 | 0.0630 | 0.72 | -0.0150 | -0.0677 | 0.0376 | 0.58 | -0.0259 | -0.0785 | 0.0268 | 0.34 |  |
| GH | -0.0241 | -0.0889 | 0.0407 | 0.47 | -0.0167 | -0.0810 | 0.0475 | 0.61 | 0.0123 | -0.0528 | 0.0774 | 0.71 |  |
| MMP1 | -0.0083 | -0.0615 | 0.0449 | 0.76 | 0.0023 | -0.0507 | 0.0554 | 0.93 | 0.0025 | -0.0503 | 0.0554 | 0.92 |  |
| RETN | 0.0190 | -0.0341 | 0.0720 | 0.48 | -0.0067 | -0.0593 | 0.0458 | 0.80 | -0.0133 | -0.0657 | 0.0391 | 0.62 |  |
| FAS | 0.0338 | -0.0206 | 0.0882 | 0.22 | 0.0103 | -0.0436 | 0.0641 | 0.71 | -0.0061 | -0.0602 | 0.0480 | 0.83 |  |
| PAPPA | -0.0236 | -0.0809 | 0.0337 | 0.42 | -0.0331 | -0.0895 | 0.0233 | 0.25 | -0.0350 | -0.0913 | 0.0213 | 0.22 |  |
| PTX3 | -0.0698 | -0.1246 | -0.0150 | 0.01 | -0.0651 | -0.1190 | -0.0111 | 0.02 | -0.0590 | -0.1128 | -0.0051 | 0.03 |  |
| REN | 0.0319 | -0.0232 | 0.0870 | 0.26 | 0.0188 | -0.0360 | 0.0736 | 0.50 | 0.0124 | -0.0423 | 0.0671 | 0.66 |  |
| CHI3L1 | -0.0195 | -0.0745 | 0.0356 | 0.49 | -0.0055 | -0.0600 | 0.0491 | 0.84 | -0.0167 | -0.0713 | 0.0379 | 0.55 |  |
| ST2 | -0.0202 | -0.0773 | 0.0369 | 0.49 | -0.0257 | -0.0821 | 0.0307 | 0.37 | -0.0306 | -0.0868 | 0.0257 | 0.29 |  |
| TIM | 0.0220 | -0.0334 | 0.0774 | 0.44 | 0.0213 | -0.0342 | 0.0768 | 0.45 | 0.0086 | -0.0469 | 0.0642 | 0.76 |  |
| mAmP | -0.0818 | -0.1413 | -0.0223 | 0.01 | -0.0603 | -0.1191 | -0.0016 | 0.04 | -0.0537 | -0.1123 | 0.0050 | 0.07 |  |
| TRANCE | -0.0227 | -0.0761 | 0.0307 | 0.41 | -0.0099 | -0.0626 | 0.0427 | 0.71 | -0.0287 | -0.0817 | 0.0243 | 0.29 |  |
| HGF | 0.0572 | 0.0025 | 0.1118 | 0.04 | 0.0280 | -0.0270 | 0.0830 | 0.32 | -0.0057 | -0.0622 | 0.0508 | 0.84 |  |
| PSGL1 | -0.0006 | -0.0623 | 0.0611 | 0.98 | -0.0061 | -0.0666 | 0.0545 | 0.84 | -0.0158 | -0.0763 | 0.0446 | 0.61 |  |
| MB | 0.0368 | -0.0201 | 0.0937 | 0.20 | 0.0183 | -0.0381 | 0.0748 | 0.52 | 0.0031 | -0.0536 | 0.0597 | 0.92 |  |
| TM | 0.0077 | -0.0469 | 0.0623 | 0.78 | -0.0153 | -0.0693 | 0.0386 | 0.58 | -0.0255 | -0.0794 | 0.0284 | 0.35 |  |
| IL16 | 0.0347 | -0.0191 | 0.0885 | 0.21 | 0.0149 | -0.0382 | 0.0681 | 0.58 | -0.0055 | -0.0591 | 0.0481 | 0.84 |  |
| MMP10 | -0.0457 | -0.0987 | 0.0073 | 0.09 | -0.0496 | -0.1032 | 0.0039 | 0.07 | -0.0391 | -0.0926 | 0.0145 | 0.15 |  |
| UPAR | 0.0136 | -0.0399 | 0.0672 | 0.62 | -0.0109 | -0.0672 | 0.0454 | 0.70 | -0.0192 | -0.0754 | 0.0371 | 0.50 |  |
| CCL4 | 0.0018 | -0.0518 | 0.0554 | 0.95 | -0.0109 | -0.0637 | 0.0419 | 0.69 | -0.0221 | -0.0749 | 0.0307 | 0.41 |  |
| CTSD | 0.0112 | -0.0439 | 0.0664 | 0.69 | 0.0091 | -0.0457 | 0.0639 | 0.74 | -0.0226 | -0.0787 | 0.0334 | 0.43 |  |
| RAGE | -0.0029 | -0.0561 | 0.0503 | 0.91 | -0.0246 | -0.0771 | 0.0280 | 0.36 | -0.0058 | -0.0587 | 0.0471 | 0.83 |  |
| CCL3 | 0.0506 | -0.0042 | 0.1053 | 0.07 | 0.0351 | -0.0191 | 0.0893 | 0.20 | 0.0143 | -0.0404 | 0.0690 | 0.61 |  |
| MMP7 | -0.0163 | -0.0703 | 0.0377 | 0.55 | -0.0267 | -0.0805 | 0.0272 | 0.33 | -0.0336 | -0.0873 | 0.0202 | 0.22 |  |
| CXCL6 | -0.0265 | -0.0794 | 0.0264 | 0.33 | -0.0279 | -0.0800 | 0.0242 | 0.29 | -0.0354 | -0.0874 | 0.0167 | 0.18 |  |
| ITGB1BP2 | -0.0598 | -0.1136 | -0.0060 | 0.03 | -0.0593 | -0.1123 | -0.0063 | 0.03 | -0.0612 | -0.1140 | -0.0083 | 0.02 |  |
| CXCL16 | 0.0092 | -0.0441 | 0.0626 | 0.73 | -0.0012 | -0.0540 | 0.0517 | 0.97 | -0.0094 | -0.0622 | 0.0433 | 0.73 |  |
| Dkk1 | 0.0125 | -0.0405 | 0.0655 | 0.64 | 0.0031 | -0.0490 | 0.0553 | 0.91 | -0.0011 | -0.0531 | 0.0510 | 0.97 |  |
| SIRT2 | -0.0079 | -0.0608 | 0.0450 | 0.77 | -0.0106 | -0.0627 | 0.0414 | 0.69 | -0.0156 | -0.0675 | 0.0363 | 0.56 |  |
| GAL | -0.0716 | -0.1262 | -0.0171 | 0.01 | -0.0743 | -0.1290 | -0.0196 | 0.01 | -0.0556 | -0.1107 | -0.0005 | 0.05 |  |
| AGRP | -0.0132 | -0.0666 | 0.0402 | 0.63 | -0.0233 | -0.0758 | 0.0293 | 0.39 | -0.0156 | -0.0681 | 0.0369 | 0.56 |  |
| CD40 | 0.0150 | -0.0379 | 0.0679 | 0.58 | 0.0057 | -0.0464 | 0.0578 | 0.83 | -0.0027 | -0.0547 | 0.0494 | 0.92 |  |
| tPA | 0.0495 | -0.0050 | 0.1040 | 0.07 | 0.0606 | 0.0068 | 0.1143 | 0.03 | 0.0340 | -0.0208 | 0.0887 | 0.22 |  |
| HBEGF | -0.0388 | -0.0919 | 0.0142 | 0.15 | -0.0448 | -0.0970 | 0.0074 | 0.09 | -0.0521 | -0.1042 | 0.0000 | 0.05 |  |
| ESM1 | -0.0344 | -0.0881 | 0.0193 | 0.21 | -0.0535 | -0.1069 | -0.0001 | 0.05 | -0.0406 | -0.0942 | 0.0129 | 0.14 |  |
| VEGFD | -0.0775 | -0.1311 | -0.0240 | 0.0046 | -0.0712 | -0.1248 | -0.0176 | 0.01 | -0.0546 | -0.1085 | -0.0008 | 0.05 |  |
| MMP12 | 0.0189 | -0.0362 | 0.0739 | 0.50 | -0.0019 | -0.0590 | 0.0553 | 0.95 | -0.0026 | -0.0596 | 0.0544 | 0.93 |  |
| SPON1 | -0.0151 | -0.0691 | 0.0390 | 0.58 | -0.0374 | -0.0909 | 0.0161 | 0.17 | -0.0374 | -0.0908 | 0.0159 | 0.17 |  |
| CASP8 | -0.0135 | -0.0686 | 0.0416 | 0.63 | -0.0146 | -0.0689 | 0.0397 | 0.60 | -0.0240 | -0.0782 | 0.0303 | 0.39 |  |
| CTSL1 | 0.0164 | -0.0380 | 0.0709 | 0.55 | -0.0116 | -0.0655 | 0.0423 | 0.67 | -0.0227 | -0.0766 | 0.0312 | 0.41 |  |
| CX3CL1 | -0.0391 | -0.0924 | 0.0142 | 0.15 | -0.0482 | -0.1006 | 0.0043 | 0.07 | -0.0477 | -0.1000 | 0.0046 | 0.07 |  |
| FABP4 | 0.0463 | -0.0116 | 0.1042 | 0.12 | 0.0277 | -0.0296 | 0.0850 | 0.34 | -0.0468 | -0.1109 | 0.0173 | 0.15 |  |
| **LEP** | **0.1604** | **0.0951** | **0.2256** | **<0.0001** | **0.1545** | **0.0894** | **0.2196** | **<0.0001** | 0.0833 | -0.0003 | 0.1669 | 0.051 |  |
| CCL20 | 0.0322 | -0.0208 | 0.0852 | 0.23 | 0.0188 | -0.0338 | 0.0715 | 0.48 | 0.0048 | -0.0481 | 0.0576 | 0.86 |  |
| CA125 | 0.0045 | -0.0510 | 0.0600 | 0.87 | 0.0059 | -0.0489 | 0.0606 | 0.83 | -0.0013 | -0.0559 | 0.0534 | 0.96 |  |
| NEMO | -0.0010 | -0.0547 | 0.0527 | 0.97 | -0.0098 | -0.0627 | 0.0430 | 0.72 | -0.0156 | -0.0684 | 0.0371 | 0.56 |  |
| FS | -0.0133 | -0.0672 | 0.0407 | 0.63 | -0.0139 | -0.0671 | 0.0392 | 0.61 | -0.0285 | -0.0818 | 0.0248 | 0.29 |  |
| PECAM1 | -0.0098 | -0.0630 | 0.0435 | 0.72 | -0.0162 | -0.0687 | 0.0362 | 0.54 | -0.0254 | -0.0778 | 0.0271 | 0.34 |  |
| NTproBNP | -0.0126 | -0.0745 | 0.0493 | 0.69 | -0.0166 | -0.0775 | 0.0443 | 0.59 | -0.0097 | -0.0705 | 0.0510 | 0.75 |  |
| ECP | 0.0485 | -0.0048 | 0.1018 | 0.07 | 0.0341 | -0.0184 | 0.0866 | 0.20 | 0.0218 | -0.0307 | 0.0744 | 0.42 |  |
| **Lipids/lipoproteins** |  |  |  |  |  |  |  |  |  |  |  |  |  |
| TC | -0.0130 | -0.0675 | 0.0415 | 0.64 | -0.0021 | -0.0558 | 0.0516 | 0.94 | -0.0126 | -0.0663 | 0.0411 | 0.65 |  |
| TG | 0.0646 | 0.0106 | 0.1185 | 0.02 | 0.0524 | -0.0011 | 0.1059 | 0.05 | 0.0158 | -0.0398 | 0.0714 | 0.58 |  |
| **HDL** | **-0.1633** | **-0.2204** | **-0.1063** | **<0.0001** | **-0.1093** | **-0.1668** | **-0.0518** | **0.0002** | -0.0736 | -0.1336 | -0.0136 | 0.02 |  |
| LDL | 0.0219 | -0.0321 | 0.0760 | 0.43 | 0.0176 | -0.0356 | 0.0708 | 0.52 | 0.0037 | -0.0496 | 0.0570 | 0.89 |  |
| small HDL | 0.0124 | -0.0449 | 0.0698 | 0.67 | 0.0256 | -0.0309 | 0.0820 | 0.37 | 0.0240 | -0.0323 | 0.0802 | 0.40 |  |
| **large HDL** | **-0.1273** | **-0.1876** | **-0.0670** | **<0.0001** | **-0.0951** | **-0.1551** | **-0.0352** | **0.0019** | -0.0633 | -0.1250 | -0.0015 | 0.04 |  |
| very small LDL | 0.0038 | -0.0545 | 0.0620 | 0.90 | 0.0060 | -0.0513 | 0.0633 | 0.84 | -0.0013 | -0.0585 | 0.0559 | 0.97 |  |
| small LDL | 0.0152 | -0.0438 | 0.0743 | 0.61 | 0.0217 | -0.0364 | 0.0797 | 0.46 | 0.0024 | -0.0561 | 0.0608 | 0.94 |  |
| medium LDL | 0.0431 | -0.0164 | 0.1025 | 0.16 | 0.0494 | -0.0092 | 0.1080 | 0.10 | 0.0232 | -0.0363 | 0.0828 | 0.44 |  |
| large LDL | 0.0726 | 0.0149 | 0.1302 | 0.01 | 0.0703 | 0.0134 | 0.1273 | 0.02 | 0.0557 | -0.0015 | 0.1128 | 0.06 |  |
| small IDL | 0.0468 | -0.0113 | 0.1048 | 0.11 | 0.0469 | -0.0103 | 0.1041 | 0.11 | 0.0338 | -0.0235 | 0.0911 | 0.25 |  |
| large IDL | -0.0196 | -0.0793 | 0.0401 | 0.52 | -0.0070 | -0.0657 | 0.0518 | 0.82 | 0.0070 | -0.0519 | 0.0658 | 0.82 |  |
| small VLDL | 0.0469 | -0.0115 | 0.1052 | 0.12 | 0.0384 | -0.0191 | 0.0959 | 0.19 | 0.0329 | -0.0244 | 0.0902 | 0.26 |  |
| medium VLDL | 0.0589 | 0.0009 | 0.1169 | 0.05 | 0.0416 | -0.0158 | 0.0990 | 0.16 | 0.0259 | -0.0317 | 0.0835 | 0.38 |  |
| large VLDL | 0.0561 | -0.0019 | 0.1142 | 0.06 | 0.0398 | -0.0177 | 0.0972 | 0.17 | 0.0213 | -0.0365 | 0.0791 | 0.47 |  |
| total VLDL | 0.0560 | -0.0021 | 0.1141 | 0.06 | 0.0424 | -0.0149 | 0.0997 | 0.15 | 0.0307 | -0.0266 | 0.0881 | 0.29 |  |
| ^a^ Using multiple linear regression analysis. Proteins, lipids, and lipoprotein subfractions were standardized as z-scores. Proteins corrected *P*<0.05/88, lipids and lipoprotein subfractions corrected *P*<0.05/16. | | | | | | | | | | | | |  |
|  |  |  |  |  |  |  |  |  |  |  |  |  |  |
| Model 1 was adjusted for age, sex, season, and total energy intake. | | | | | | | | | | | | |  |
| Model 2 was adjusted for age, sex, season, total energy intake, education, smoking status, alcohol consumption, and leisure-time physical activity. | | | | | | | | | | | | |  |
| Model 3 was further adjusted for body mass index. | | | | | | | | | | | | |  |

| **Supplementary Table 2.** Plasma proteins, lipids, and lipoprotein subfractions associated with fermented milk intake ^a^ | | | | | | | | | | | | |  |
| --- | --- | --- | --- | --- | --- | --- | --- | --- | --- | --- | --- | --- | --- |
|  | **Model 1** | | | | **Model 2** | | | | **Model 3** | | | |  |
|  | *β* | 95% CI | | *P* value | *β* | 95% CI | | *P* value | *β* | 95% CI | | *P* value |  |
| **Plasma proteins** |  |  |  |  |  |  |  |  |  |  |  |  |  |
| IL8 | -0.2367 | -0.3783 | -0.0950 | 0.0011 | -0.1493 | -0.2910 | -0.0075 | 0.04 | -0.1495 | -0.2913 | -0.0077 | 0.04 |  |
| VEGFA | -0.1223 | -0.2636 | 0.0190 | 0.09 | -0.0417 | -0.1828 | 0.0993 | 0.56 | -0.0438 | -0.1860 | 0.0985 | 0.55 |  |
| AM | -0.1501 | -0.2950 | -0.0053 | 0.04 | -0.0766 | -0.2210 | 0.0678 | 0.30 | -0.0851 | -0.2346 | 0.0645 | 0.26 |  |
| CD40L | -0.1409 | -0.2807 | -0.0010 | 0.05 | -0.0948 | -0.2336 | 0.0441 | 0.18 | -0.0952 | -0.2342 | 0.0438 | 0.18 |  |
| GDF15 | -0.2274 | -0.3772 | -0.0776 | 0.0029 | -0.0398 | -0.1957 | 0.1161 | 0.62 | -0.0406 | -0.1969 | 0.1156 | 0.61 |  |
| PlGF | -0.0789 | -0.2253 | 0.0674 | 0.29 | -0.0243 | -0.1698 | 0.1213 | 0.74 | -0.0255 | -0.1717 | 0.1207 | 0.73 |  |
| SELE | -0.0349 | -0.1780 | 0.1081 | 0.63 | 0.0159 | -0.1260 | 0.1578 | 0.83 | 0.0145 | -0.1301 | 0.1591 | 0.84 |  |
| EGF | -0.1052 | -0.2451 | 0.0346 | 0.14 | -0.0747 | -0.2134 | 0.0639 | 0.29 | -0.0751 | -0.2138 | 0.0636 | 0.29 |  |
| OPG | -0.0798 | -0.2248 | 0.0651 | 0.28 | -0.0248 | -0.1689 | 0.1194 | 0.74 | -0.0249 | -0.1691 | 0.1192 | 0.73 |  |
| SRC | 0.1021 | -0.0390 | 0.2431 | 0.16 | 0.0666 | -0.0731 | 0.2064 | 0.35 | 0.0668 | -0.0730 | 0.2065 | 0.35 |  |
| IL1ra | -0.0972 | -0.2493 | 0.0549 | 0.21 | -0.0582 | -0.2102 | 0.0939 | 0.45 | -0.0733 | -0.2348 | 0.0881 | 0.37 |  |
| IL6 | -0.2211 | -0.3629 | -0.0794 | 0.002 | -0.1003 | -0.2439 | 0.0433 | 0.17 | -0.1050 | -0.2507 | 0.0407 | 0.16 |  |
| CSTB | -0.1915 | -0.3345 | -0.0486 | 0.009 | -0.0913 | -0.2347 | 0.0520 | 0.21 | -0.0961 | -0.2418 | 0.0495 | 0.20 |  |
| MCP1 | -0.1082 | -0.2503 | 0.0340 | 0.14 | -0.0536 | -0.1948 | 0.0876 | 0.46 | -0.0546 | -0.1962 | 0.0870 | 0.45 |  |
| KLK6 | 0.0071 | -0.1341 | 0.1482 | 0.92 | 0.0034 | -0.1368 | 0.1436 | 0.96 | 0.0043 | -0.1365 | 0.1450 | 0.95 |  |
| Gal3 | 0.0262 | -0.1170 | 0.1694 | 0.72 | 0.0340 | -0.1081 | 0.1761 | 0.64 | 0.0334 | -0.1091 | 0.1759 | 0.65 |  |
| PAR1 | -0.0818 | -0.2224 | 0.0588 | 0.25 | -0.0484 | -0.1877 | 0.0909 | 0.50 | -0.0487 | -0.1880 | 0.0906 | 0.49 |  |
| TRAIL | -0.0434 | -0.1844 | 0.0975 | 0.55 | 0.0138 | -0.1263 | 0.1539 | 0.85 | 0.0128 | -0.1284 | 0.1539 | 0.86 |  |
| hK11 | 0.0338 | -0.1078 | 0.1755 | 0.64 | 0.0420 | -0.0982 | 0.1822 | 0.56 | 0.0426 | -0.0978 | 0.1829 | 0.55 |  |
| TIE2 | -0.0487 | -0.1900 | 0.0926 | 0.50 | -0.0343 | -0.1743 | 0.1057 | 0.63 | -0.0352 | -0.1756 | 0.1052 | 0.62 |  |
| TF | 0.0106 | -0.1321 | 0.1534 | 0.88 | 0.0250 | -0.1164 | 0.1665 | 0.73 | 0.0254 | -0.1162 | 0.1669 | 0.73 |  |
| TNFR1 | -0.1042 | -0.2486 | 0.0402 | 0.16 | -0.0320 | -0.1763 | 0.1123 | 0.66 | -0.0347 | -0.1810 | 0.1117 | 0.64 |  |
| PDGFsubunitB | -0.1429 | -0.2832 | -0.0027 | 0.05 | -0.1003 | -0.2395 | 0.0389 | 0.16 | -0.1009 | -0.2402 | 0.0385 | 0.16 |  |
| IL27A | 0.0709 | -0.0703 | 0.2120 | 0.32 | 0.0311 | -0.1098 | 0.1720 | 0.67 | 0.0313 | -0.1097 | 0.1723 | 0.66 |  |
| CSF1 | -0.0601 | -0.2013 | 0.0812 | 0.40 | -0.0101 | -0.1509 | 0.1307 | 0.89 | -0.0117 | -0.1539 | 0.1305 | 0.87 |  |
| CXCL1 | -0.0632 | -0.2031 | 0.0766 | 0.38 | -0.0098 | -0.1489 | 0.1292 | 0.89 | -0.0101 | -0.1491 | 0.1290 | 0.89 |  |
| LOX1 | -0.1166 | -0.2571 | 0.0239 | 0.10 | 0.0123 | -0.1304 | 0.1549 | 0.87 | 0.0120 | -0.1307 | 0.1547 | 0.87 |  |
| TRAILR2 | -0.2074 | -0.3507 | -0.0642 | 0.0045 | -0.0689 | -0.2151 | 0.0772 | 0.36 | -0.0708 | -0.2178 | 0.0762 | 0.35 |  |
| FGF23 | -0.1694 | -0.3093 | -0.0295 | 0.02 | -0.1384 | -0.2770 | 0.0003 | 0.05 | -0.1416 | -0.2813 | -0.0019 | 0.05 |  |
| SCF | 0.1236 | -0.0172 | 0.2643 | 0.09 | 0.0451 | -0.0980 | 0.1882 | 0.54 | 0.0466 | -0.0972 | 0.1905 | 0.53 |  |
| **IL18** | **-0.2552** | **-0.3992** | **-0.1112** | **0.00052** | -0.1833 | -0.3269 | -0.0396 | 0.012 | -0.1868 | -0.3314 | -0.0422 | 0.01 |  |
| IL6RA | -0.0005 | -0.1407 | 0.1398 | 0.99 | 0.0233 | -0.1157 | 0.1624 | 0.74 | 0.0227 | -0.1167 | 0.1622 | 0.75 |  |
| TNFR2 | -0.0983 | -0.2411 | 0.0444 | 0.18 | -0.0196 | -0.1626 | 0.1234 | 0.79 | -0.0210 | -0.1649 | 0.1230 | 0.78 |  |
| MMP3 | -0.1100 | -0.2835 | 0.0634 | 0.21 | -0.0890 | -0.2607 | 0.0826 | 0.31 | -0.0889 | -0.2606 | 0.0828 | 0.31 |  |
| HSP27 | -0.0938 | -0.2340 | 0.0465 | 0.19 | -0.0855 | -0.2243 | 0.0533 | 0.23 | -0.0861 | -0.2250 | 0.0529 | 0.22 |  |
| TNFSF14 | -0.1231 | -0.2632 | 0.0169 | 0.08 | -0.0536 | -0.1932 | 0.0860 | 0.45 | -0.0554 | -0.1958 | 0.0850 | 0.44 |  |
| PRL | 0.0635 | -0.0791 | 0.2062 | 0.38 | -0.0030 | -0.1450 | 0.1389 | 0.97 | -0.0028 | -0.1448 | 0.1392 | 0.97 |  |
| MPO | -0.1347 | -0.2753 | 0.0059 | 0.06 | -0.0765 | -0.2165 | 0.0635 | 0.28 | -0.0779 | -0.2184 | 0.0627 | 0.28 |  |
| GH | 0.1121 | -0.0592 | 0.2835 | 0.20 | 0.1380 | -0.0330 | 0.3089 | 0.11 | 0.1446 | -0.0290 | 0.3183 | 0.10 |  |
| MMP1 | -0.2306 | -0.3711 | -0.0900 | 0.0013 | -0.1597 | -0.3007 | -0.0186 | 0.03 | -0.1596 | -0.3007 | -0.0186 | 0.03 |  |
| RETN | -0.1136 | -0.2539 | 0.0267 | 0.11 | -0.0556 | -0.1953 | 0.0841 | 0.44 | -0.0562 | -0.1961 | 0.0837 | 0.43 |  |
| FAS | 0.0087 | -0.1351 | 0.1526 | 0.91 | 0.0808 | -0.0625 | 0.2240 | 0.27 | 0.0807 | -0.0636 | 0.2250 | 0.27 |  |
| PAPPA | 0.0132 | -0.1386 | 0.1650 | 0.86 | 0.0030 | -0.1475 | 0.1534 | 0.97 | 0.0027 | -0.1477 | 0.1532 | 0.97 |  |
| PTX3 | -0.0093 | -0.1537 | 0.1350 | 0.90 | -0.0113 | -0.1542 | 0.1316 | 0.88 | -0.0110 | -0.1541 | 0.1321 | 0.88 |  |
| REN | -0.1166 | -0.2621 | 0.0290 | 0.12 | -0.0220 | -0.1678 | 0.1239 | 0.77 | -0.0225 | -0.1685 | 0.1235 | 0.76 |  |
| CHI3L1 | -0.0629 | -0.2084 | 0.0826 | 0.40 | 0.0009 | -0.1442 | 0.1460 | 0.99 | 0.0000 | -0.1456 | 0.1456 | 1.00 |  |
| ST2 | 0.0203 | -0.1307 | 0.1714 | 0.79 | 0.0090 | -0.1410 | 0.1590 | 0.91 | 0.0087 | -0.1415 | 0.1588 | 0.91 |  |
| TIM | -0.0938 | -0.2402 | 0.0526 | 0.21 | 0.0296 | -0.1180 | 0.1772 | 0.69 | 0.0288 | -0.1194 | 0.1771 | 0.70 |  |
| mAmP | 0.1328 | -0.0255 | 0.2911 | 0.10 | 0.1284 | -0.0291 | 0.2858 | 0.11 | 0.1322 | -0.0254 | 0.2899 | 0.10 |  |
| TRANCE | 0.0299 | -0.1115 | 0.1714 | 0.68 | -0.0033 | -0.1436 | 0.1370 | 0.96 | -0.0027 | -0.1445 | 0.1392 | 0.97 |  |
| HGF | -0.1649 | -0.3093 | -0.0205 | 0.03 | -0.0372 | -0.1836 | 0.1091 | 0.62 | -0.0422 | -0.1930 | 0.1087 | 0.58 |  |
| PSGL1 | -0.1178 | -0.2781 | 0.0424 | 0.15 | -0.1201 | -0.2782 | 0.0381 | 0.14 | -0.1234 | -0.2819 | 0.0351 | 0.13 |  |
| MB | 0.0496 | -0.1008 | 0.2000 | 0.52 | 0.0083 | -0.1419 | 0.1586 | 0.91 | 0.0073 | -0.1438 | 0.1584 | 0.92 |  |
| TM | -0.0502 | -0.1945 | 0.0942 | 0.50 | -0.0280 | -0.1715 | 0.1154 | 0.70 | -0.0289 | -0.1728 | 0.1149 | 0.69 |  |
| IL16 | 0.0080 | -0.1343 | 0.1502 | 0.91 | 0.0474 | -0.0940 | 0.1888 | 0.51 | 0.0470 | -0.0961 | 0.1901 | 0.52 |  |
| MMP10 | -0.1264 | -0.2665 | 0.0137 | 0.08 | -0.0189 | -0.1614 | 0.1236 | 0.79 | -0.0182 | -0.1612 | 0.1248 | 0.80 |  |
| **UPAR** | **-0.2752** | **-0.4166** | **-0.1338** | **0.00014** | -0.0871 | -0.2369 | 0.0627 | 0.25 | -0.0880 | -0.2381 | 0.0621 | 0.25 |  |
| CCL4 | -0.1036 | -0.2452 | 0.0380 | 0.15 | -0.0638 | -0.2043 | 0.0767 | 0.37 | -0.0650 | -0.2060 | 0.0759 | 0.37 |  |
| CTSD | -0.2307 | -0.3764 | -0.0850 | 0.0019 | -0.1380 | -0.2838 | 0.0077 | 0.06 | -0.1476 | -0.2972 | 0.0019 | 0.05 |  |
| RAGE | 0.0336 | -0.1070 | 0.1743 | 0.64 | 0.0396 | -0.1002 | 0.1793 | 0.58 | 0.0419 | -0.0994 | 0.1831 | 0.56 |  |
| CCL3 | -0.1984 | -0.3425 | -0.0543 | 0.01 | -0.1543 | -0.2976 | -0.0109 | 0.03 | -0.1598 | -0.3050 | -0.0146 | 0.03 |  |
| MMP7 | -0.1647 | -0.3074 | -0.0219 | 0.02 | -0.0787 | -0.2220 | 0.0646 | 0.28 | -0.0795 | -0.2230 | 0.0640 | 0.28 |  |
| CXCL6 | -0.0915 | -0.2314 | 0.0485 | 0.20 | -0.0802 | -0.2187 | 0.0584 | 0.26 | -0.0810 | -0.2198 | 0.0579 | 0.25 |  |
| ITGB1BP2 | -0.1771 | -0.3198 | -0.0345 | 0.01 | -0.1394 | -0.2807 | 0.0020 | 0.05 | -0.1398 | -0.2811 | 0.0016 | 0.05 |  |
| CXCL16 | -0.0196 | -0.1605 | 0.1214 | 0.79 | 0.0468 | -0.0935 | 0.1872 | 0.51 | 0.0460 | -0.0946 | 0.1866 | 0.52 |  |
| Dkk1 | -0.0710 | -0.2112 | 0.0691 | 0.32 | -0.0535 | -0.1923 | 0.0853 | 0.45 | -0.0539 | -0.1928 | 0.0850 | 0.45 |  |
| SIRT2 | -0.1275 | -0.2673 | 0.0123 | 0.07 | -0.1000 | -0.2384 | 0.0384 | 0.16 | -0.1005 | -0.2391 | 0.0380 | 0.15 |  |
| GAL | 0.0918 | -0.0526 | 0.2363 | 0.21 | -0.0072 | -0.1528 | 0.1383 | 0.92 | -0.0059 | -0.1530 | 0.1413 | 0.94 |  |
| AGRP | -0.0144 | -0.1556 | 0.1268 | 0.84 | -0.0111 | -0.1509 | 0.1288 | 0.88 | -0.0105 | -0.1506 | 0.1296 | 0.88 |  |
| CD40 | 0.0080 | -0.1319 | 0.1479 | 0.91 | 0.0318 | -0.1068 | 0.1704 | 0.65 | 0.0313 | -0.1076 | 0.1702 | 0.66 |  |
| tPA | 0.1045 | -0.0396 | 0.2487 | 0.16 | 0.1184 | -0.0245 | 0.2614 | 0.10 | 0.1216 | -0.0246 | 0.2677 | 0.10 |  |
| HBEGF | -0.0905 | -0.2308 | 0.0498 | 0.21 | -0.0787 | -0.2176 | 0.0602 | 0.27 | -0.0795 | -0.2186 | 0.0596 | 0.26 |  |
| ESM1 | 0.1998 | 0.0579 | 0.3417 | 0.01 | 0.1449 | 0.0028 | 0.2869 | 0.05 | 0.1473 | 0.0045 | 0.2901 | 0.04 |  |
| VEGFD | -0.1306 | -0.2723 | 0.0111 | 0.07 | -0.0697 | -0.2123 | 0.0729 | 0.34 | -0.0695 | -0.2133 | 0.0743 | 0.34 |  |
| **MMP12** | **-0.2602** | **-0.4055** | **-0.1148** | **0.00045** | -0.0858 | -0.2378 | 0.0663 | 0.27 | -0.0858 | -0.2379 | 0.0663 | 0.27 |  |
| SPON1 | -0.0869 | -0.2298 | 0.0559 | 0.23 | -0.0163 | -0.1587 | 0.1261 | 0.82 | -0.0163 | -0.1587 | 0.1261 | 0.82 |  |
| CASP8 | -0.1668 | -0.3119 | -0.0217 | 0.02 | -0.1307 | -0.2745 | 0.0132 | 0.07 | -0.1314 | -0.2756 | 0.0128 | 0.07 |  |
| CTSL1 | -0.0615 | -0.2054 | 0.0825 | 0.40 | 0.0000 | -0.1434 | 0.1434 | 1.00 | -0.0008 | -0.1447 | 0.1431 | 0.99 |  |
| CX3CL1 | -0.0575 | -0.1984 | 0.0834 | 0.42 | -0.0637 | -0.2033 | 0.0760 | 0.37 | -0.0637 | -0.2033 | 0.0760 | 0.37 |  |
| FABP4 | -0.0246 | -0.1778 | 0.1286 | 0.75 | 0.0116 | -0.1410 | 0.1642 | 0.88 | 0.0101 | -0.1611 | 0.1814 | 0.91 |  |
| LEP | 0.0654 | -0.1073 | 0.2381 | 0.46 | 0.0399 | -0.1333 | 0.2131 | 0.65 | 0.0658 | -0.1568 | 0.2883 | 0.56 |  |
| CCL20 | -0.1240 | -0.2640 | 0.0160 | 0.08 | -0.0357 | -0.1758 | 0.1045 | 0.62 | -0.0371 | -0.1781 | 0.1038 | 0.61 |  |
| CA125 | 0.0131 | -0.1338 | 0.1601 | 0.86 | -0.0063 | -0.1520 | 0.1393 | 0.93 | -0.0083 | -0.1542 | 0.1376 | 0.91 |  |
| NEMO | 0.0700 | -0.0719 | 0.2120 | 0.33 | 0.0746 | -0.0660 | 0.2153 | 0.30 | 0.0743 | -0.0664 | 0.2151 | 0.30 |  |
| FS | -0.0561 | -0.1986 | 0.0864 | 0.44 | -0.0031 | -0.1446 | 0.1384 | 0.97 | -0.0042 | -0.1465 | 0.1381 | 0.95 |  |
| PECAM1 | 0.0567 | -0.0842 | 0.1976 | 0.43 | 0.0630 | -0.0766 | 0.2025 | 0.38 | 0.0626 | -0.0774 | 0.2025 | 0.38 |  |
| NTproBNP | -0.1443 | -0.3022 | 0.0136 | 0.07 | -0.1317 | -0.2881 | 0.0247 | 0.10 | -0.1287 | -0.2853 | 0.0279 | 0.11 |  |
| ECP | 0.0299 | -0.1110 | 0.1707 | 0.68 | 0.0471 | -0.0926 | 0.1867 | 0.51 | 0.0467 | -0.0937 | 0.1870 | 0.51 |  |
| **Lipids/lipoproteins** |  |  |  |  |  |  |  |  |  |  |  |  |  |
| TC | -0.0686 | -0.2126 | 0.0755 | 0.35 | -0.0464 | -0.1891 | 0.0964 | 0.52 | -0.0474 | -0.1906 | 0.0958 | 0.52 |  |
| TG | -0.1506 | -0.2933 | -0.0080 | 0.04 | -0.0804 | -0.2228 | 0.0619 | 0.27 | -0.0901 | -0.2384 | 0.0583 | 0.23 |  |
| HDL | 0.1776 | 0.0262 | 0.3289 | 0.02 | 0.0895 | -0.0639 | 0.2428 | 0.25 | 0.1002 | -0.0601 | 0.2605 | 0.22 |  |
| LDL | -0.0956 | -0.2384 | 0.0472 | 0.19 | -0.0609 | -0.2023 | 0.0806 | 0.40 | -0.0622 | -0.2045 | 0.0801 | 0.39 |  |
| small HDL | -0.0961 | -0.2462 | 0.0539 | 0.21 | -0.0945 | -0.2430 | 0.0541 | 0.21 | -0.0944 | -0.2430 | 0.0541 | 0.21 |  |
| large HDL | 0.0858 | -0.0724 | 0.2439 | 0.29 | 0.0098 | -0.1483 | 0.1678 | 0.90 | 0.0091 | -0.1542 | 0.1723 | 0.91 |  |
| very small LDL | -0.1493 | -0.3017 | 0.0030 | 0.05 | -0.1378 | -0.2886 | 0.0129 | 0.07 | -0.1380 | -0.2890 | 0.0130 | 0.07 |  |
| small LDL | -0.1987 | -0.3531 | -0.0444 | 0.01 | -0.1870 | -0.3397 | -0.0342 | 0.02 | -0.1900 | -0.3443 | -0.0357 | 0.02 |  |
| medium LDL | -0.1957 | -0.3511 | -0.0402 | 0.01 | -0.1604 | -0.3145 | -0.0063 | 0.04 | -0.1657 | -0.3228 | -0.0085 | 0.04 |  |
| large LDL | -0.1213 | -0.2724 | 0.0297 | 0.12 | -0.0578 | -0.2078 | 0.0922 | 0.45 | -0.0580 | -0.2090 | 0.0930 | 0.45 |  |
| small IDL | -0.1716 | -0.3234 | -0.0197 | 0.03 | -0.1452 | -0.2956 | 0.0053 | 0.06 | -0.1461 | -0.2973 | 0.0052 | 0.06 |  |
| large IDL | 0.0054 | -0.1508 | 0.1617 | 0.95 | -0.0063 | -0.1609 | 0.1484 | 0.94 | -0.0068 | -0.1622 | 0.1486 | 0.93 |  |
| small VLDL | -0.1554 | -0.3081 | -0.0028 | 0.05 | -0.1269 | -0.2781 | 0.0243 | 0.10 | -0.1269 | -0.2783 | 0.0244 | 0.10 |  |
| **medium VLDL** | **-0.2347** | **-0.3865** | **-0.0830** | **0.0024** | -0.1732 | -0.3243 | -0.0222 | 0.02 | -0.1751 | -0.3272 | -0.0229 | 0.02 |  |
| **large VLDL** | **-0.2513** | **-0.4030** | **-0.0996** | **0.0012** | -0.1918 | -0.3428 | -0.0407 | 0.01 | -0.1948 | -0.3473 | -0.0422 | 0.01 |  |
| total VLDL | -0.2102 | -0.3621 | -0.0583 | 0.01 | -0.1624 | -0.3132 | -0.0115 | 0.03 | -0.1632 | -0.3146 | -0.0117 | 0.03 |  |
| ^a^ Using multiple linear regression analysis. Proteins, lipids, and lipoprotein subfractions were standardized as z-scores. Proteins corrected *P*<0.05/88, lipids and lipoprotein subfractions corrected *P*<0.05/16. | | | | | | | | | | | | |  |
|  |  |  |  |  |  |  |  |  |  |  |  |  |  |
| Model 1 was adjusted for age, sex, season, and total energy intake. | | | | | | | | | | | | |  |
| Model 2 was adjusted for age, sex, season, total energy intake, education, smoking status, alcohol consumption, and leisure-time physical activity. | | | | | | | | | | | | |  |
| Model 3 was further adjusted for body mass index. | | | | | | | | | | | | |  |

| **Supplementary Table 3.** Plasma proteins, lipids, and lipoprotein subfractions associated with the lactase persistent genetic variant ^a^ | | | | | | | | | |  |
| --- | --- | --- | --- | --- | --- | --- | --- | --- | --- | --- |
|  | Logistic regression model ^b^ | | | |  | Linear regression model ^c^ | | | |  |
|  | OR | 95% CI | | *P* value |  | *β* | 95% CI | | *P* value |  |
| **Plasma proteins** |  |  |  |  |  |  |  |  |  |  |
| IL8 | 0.94 | 0.79 | 1.12 | 0.49 |  | 0.0047 | -0.0143 | 0.0237 | 0.63 |  |
| VEGFA | 0.84 | 0.71 | 0.99 | 0.04 |  | 0.0101 | -0.0088 | 0.0291 | 0.29 |  |
| AM | 0.85 | 0.72 | 1.00 | 0.051 |  | 0.0226 | 0.0033 | 0.0418 | 0.02 |  |
| CD40L | 0.88 | 0.75 | 1.04 | 0.13 |  | 0.0094 | -0.0093 | 0.0282 | 0.32 |  |
| GDF15 | 0.86 | 0.72 | 1.04 | 0.12 |  | 0.0159 | -0.0042 | 0.0361 | 0.12 |  |
| PlGF | 0.87 | 0.74 | 1.03 | 0.11 |  | 0.0164 | -0.0032 | 0.0360 | 0.10 |  |
| SELE | 0.99 | 0.84 | 1.18 | 0.95 |  | 0.0073 | -0.0119 | 0.0265 | 0.46 |  |
| EGF | 0.85 | 0.72 | 1.00 | 0.05 |  | 0.0080 | -0.0108 | 0.0268 | 0.41 |  |
| OPG | 0.94 | 0.79 | 1.11 | 0.46 |  | 0.0000 | -0.0195 | 0.0195 | 1.00 |  |
| SRC | 1.06 | 0.90 | 1.26 | 0.48 |  | 0.0005 | -0.0182 | 0.0193 | 0.95 |  |
| IL1ra | 0.92 | 0.76 | 1.11 | 0.36 |  | 0.0199 | -0.0003 | 0.0402 | 0.054 |  |
| IL6 | 0.92 | 0.77 | 1.09 | 0.33 |  | -0.0008 | -0.0199 | 0.0183 | 0.93 |  |
| CSTB | 0.86 | 0.73 | 1.02 | 0.09 |  | 0.0140 | -0.0052 | 0.0332 | 0.15 |  |
| MCP1 | 0.91 | 0.76 | 1.07 | 0.25 |  | 0.0081 | -0.0109 | 0.0271 | 0.40 |  |
| KLK6 | 0.98 | 0.83 | 1.16 | 0.85 |  | 0.0062 | -0.0127 | 0.0252 | 0.52 |  |
| Gal3 | 0.97 | 0.82 | 1.15 | 0.76 |  | 0.0043 | -0.0149 | 0.0235 | 0.66 |  |
| PAR1 | 0.91 | 0.78 | 1.07 | 0.27 |  | 0.0014 | -0.0174 | 0.0202 | 0.89 |  |
| TRAIL | 0.95 | 0.80 | 1.11 | 0.50 |  | 0.0080 | -0.0109 | 0.0268 | 0.41 |  |
| hK11 | 0.91 | 0.77 | 1.08 | 0.27 |  | 0.0084 | -0.0105 | 0.0274 | 0.38 |  |
| TIE2 | 0.95 | 0.81 | 1.12 | 0.57 |  | 0.0028 | -0.0161 | 0.0217 | 0.77 |  |
| TF | 0.99 | 0.84 | 1.17 | 0.93 |  | 0.0062 | -0.0129 | 0.0254 | 0.52 |  |
| TNFR1 | 0.86 | 0.73 | 1.02 | 0.08 |  | 0.0159 | -0.0034 | 0.0353 | 0.11 |  |
| PDGFsubunitB | 0.87 | 0.74 | 1.02 | 0.09 |  | 0.0049 | -0.0139 | 0.0238 | 0.61 |  |
| IL27A | 1.06 | 0.90 | 1.25 | 0.48 |  | -0.0103 | -0.0292 | 0.0086 | 0.29 |  |
| CSF1 | 0.96 | 0.81 | 1.13 | 0.61 |  | 0.0020 | -0.0168 | 0.0209 | 0.83 |  |
| CXCL1 | 0.88 | 0.75 | 1.04 | 0.13 |  | 0.0011 | -0.0177 | 0.0199 | 0.91 |  |
| LOX1 | 1.00 | 0.85 | 1.18 | 0.98 |  | 0.0010 | -0.0179 | 0.0198 | 0.92 |  |
| TRAILR2 | 0.90 | 0.75 | 1.08 | 0.26 |  | 0.0161 | -0.0031 | 0.0352 | 0.10 |  |
| FGF23 | 0.88 | 0.74 | 1.05 | 0.16 |  | 0.0035 | -0.0153 | 0.0223 | 0.72 |  |
| SCF | 1.03 | 0.87 | 1.22 | 0.73 |  | -0.0105 | -0.0293 | 0.0084 | 0.28 |  |
| IL18 | 0.92 | 0.78 | 1.09 | 0.36 |  | 0.0074 | -0.0119 | 0.0267 | 0.45 |  |
| IL6RA | 0.96 | 0.81 | 1.13 | 0.62 |  | 0.0010 | -0.0178 | 0.0199 | 0.91 |  |
| TNFR2 | 0.93 | 0.78 | 1.10 | 0.37 |  | 0.0116 | -0.0076 | 0.0307 | 0.24 |  |
| MMP3 | 1.04 | 0.85 | 1.28 | 0.70 |  | 0.0166 | -0.0068 | 0.0399 | 0.16 |  |
| HSP27 | 0.85 | 0.73 | 1.00 | 0.05 |  | 0.0114 | -0.0075 | 0.0302 | 0.24 |  |
| TNFSF14 | 0.86 | 0.73 | 1.02 | 0.08 |  | 0.0159 | -0.0029 | 0.0347 | 0.10 |  |
| PRL | 0.91 | 0.76 | 1.07 | 0.26 |  | 0.0033 | -0.0158 | 0.0225 | 0.73 |  |
| MPO | 1.07 | 0.91 | 1.27 | 0.41 |  | -0.0058 | -0.0247 | 0.0131 | 0.55 |  |
| GH | 0.94 | 0.77 | 1.15 | 0.55 |  | -0.0185 | -0.0416 | 0.0045 | 0.12 |  |
| MMP1 | 0.80 | 0.68 | 0.95 | 0.01 |  | 0.0084 | -0.0105 | 0.0272 | 0.38 |  |
| RETN | 0.88 | 0.74 | 1.04 | 0.13 |  | 0.0128 | -0.0060 | 0.0317 | 0.18 |  |
| FAS | 0.95 | 0.80 | 1.13 | 0.55 |  | 0.0056 | -0.0137 | 0.0249 | 0.57 |  |
| PAPPA | 1.12 | 0.94 | 1.33 | 0.22 |  | -0.0054 | -0.0257 | 0.0149 | 0.60 |  |
| PTX3 | 0.96 | 0.81 | 1.13 | 0.61 |  | 0.0031 | -0.0163 | 0.0225 | 0.76 |  |
| REN | 0.95 | 0.80 | 1.13 | 0.56 |  | 0.0098 | -0.0098 | 0.0294 | 0.33 |  |
| CHI3L1 | 1.10 | 0.93 | 1.30 | 0.27 |  | 0.0037 | -0.0158 | 0.0232 | 0.71 |  |
| ST2 | 0.99 | 0.83 | 1.18 | 0.93 |  | 0.0047 | -0.0155 | 0.0249 | 0.65 |  |
| TIM | 0.80 | 0.67 | 0.95 | 0.01 |  | 0.0185 | -0.0012 | 0.0381 | 0.07 |  |
| mAmP | 0.99 | 0.83 | 1.19 | 0.91 |  | -0.0078 | -0.0294 | 0.0137 | 0.48 |  |
| TRANCE | 0.99 | 0.84 | 1.16 | 0.89 |  | 0.0039 | -0.0151 | 0.0228 | 0.69 |  |
| HGF | 0.88 | 0.74 | 1.04 | 0.14 |  | 0.0135 | -0.0058 | 0.0328 | 0.17 |  |
| PSGL1 | 1.09 | 0.92 | 1.31 | 0.32 |  | 0.0086 | -0.0129 | 0.0300 | 0.43 |  |
| MB | 0.91 | 0.76 | 1.09 | 0.30 |  | 0.0040 | -0.0162 | 0.0242 | 0.70 |  |
| TM | 0.94 | 0.80 | 1.11 | 0.49 |  | 0.0010 | -0.0183 | 0.0203 | 0.92 |  |
| IL16 | 0.95 | 0.81 | 1.12 | 0.55 |  | -0.0012 | -0.0202 | 0.0179 | 0.90 |  |
| MMP10 | 1.01 | 0.86 | 1.19 | 0.88 |  | 0.0061 | -0.0127 | 0.0249 | 0.52 |  |
| UPAR | 0.99 | 0.84 | 1.17 | 0.91 |  | 0.0010 | -0.0180 | 0.0200 | 0.92 |  |
| CCL4 | 0.87 | 0.73 | 1.04 | 0.14 |  | -0.0020 | -0.0210 | 0.0170 | 0.84 |  |
| CTSD | 0.96 | 0.81 | 1.14 | 0.66 |  | 0.0149 | -0.0047 | 0.0345 | 0.14 |  |
| RAGE | 0.95 | 0.81 | 1.12 | 0.57 |  | 0.0048 | -0.0141 | 0.0236 | 0.62 |  |
| CCL3 | 0.96 | 0.81 | 1.15 | 0.68 |  | 0.0086 | -0.0107 | 0.0279 | 0.38 |  |
| MMP7 | 1.00 | 0.85 | 1.19 | 0.98 |  | 0.0022 | -0.0169 | 0.0214 | 0.82 |  |
| CXCL6 | 0.85 | 0.72 | 1.01 | 0.06 |  | 0.0127 | -0.0061 | 0.0315 | 0.18 |  |
| ITGB1BP2 | 0.92 | 0.77 | 1.09 | 0.33 |  | 0.0045 | -0.0146 | 0.0236 | 0.64 |  |
| CXCL16 | 1.02 | 0.86 | 1.20 | 0.83 |  | 0.0029 | -0.0160 | 0.0218 | 0.77 |  |
| Dkk1 | 0.86 | 0.73 | 1.01 | 0.07 |  | 0.0051 | -0.0137 | 0.0239 | 0.60 |  |
| SIRT2 | 0.86 | 0.73 | 1.02 | 0.08 |  | 0.0092 | -0.0096 | 0.0280 | 0.34 |  |
| GAL | 1.03 | 0.87 | 1.23 | 0.70 |  | -0.0034 | -0.0229 | 0.0160 | 0.73 |  |
| AGRP | 1.01 | 0.86 | 1.20 | 0.88 |  | 0.0082 | -0.0107 | 0.0272 | 0.40 |  |
| CD40 | 0.96 | 0.81 | 1.13 | 0.59 |  | 0.0075 | -0.0113 | 0.0263 | 0.43 |  |
| tPA | 0.98 | 0.83 | 1.17 | 0.86 |  | 0.0126 | -0.0068 | 0.0319 | 0.20 |  |
| HBEGF | 0.96 | 0.82 | 1.14 | 0.66 |  | -0.0033 | -0.0222 | 0.0155 | 0.73 |  |
| ESM1 | 1.13 | 0.96 | 1.33 | 0.15 |  | -0.0100 | -0.0291 | 0.0091 | 0.30 |  |
| VEGFD | 1.18 | 1.00 | 1.40 | 0.06 |  | -0.0091 | -0.0281 | 0.0100 | 0.35 |  |
| MMP12 | 0.92 | 0.77 | 1.09 | 0.35 |  | 0.0023 | -0.0173 | 0.0219 | 0.82 |  |
| SPON1 | 1.00 | 0.85 | 1.18 | 0.99 |  | 0.0045 | -0.0146 | 0.0237 | 0.64 |  |
| CASP8 | 0.91 | 0.76 | 1.08 | 0.27 |  | 0.0039 | -0.0153 | 0.0232 | 0.69 |  |
| CTSL1 | 0.95 | 0.80 | 1.13 | 0.58 |  | -0.0054 | -0.0247 | 0.0140 | 0.59 |  |
| CX3CL1 | 1.03 | 0.87 | 1.21 | 0.76 |  | -0.0018 | -0.0207 | 0.0171 | 0.85 |  |
| FABP4 | 1.01 | 0.84 | 1.21 | 0.90 |  | 0.0086 | -0.0119 | 0.0291 | 0.41 |  |
| LEP | 0.87 | 0.72 | 1.06 | 0.17 |  | 0.0297 | 0.0065 | 0.0529 | 0.01 |  |
| CCL20 | 1.03 | 0.88 | 1.22 | 0.68 |  | -0.0043 | -0.0231 | 0.0145 | 0.66 |  |
| CA125 | 1.12 | 0.95 | 1.33 | 0.17 |  | -0.0049 | -0.0245 | 0.0148 | 0.63 |  |
| NEMO | 1.01 | 0.86 | 1.19 | 0.88 |  | 0.0032 | -0.0156 | 0.0220 | 0.74 |  |
| FS | 0.85 | 0.72 | 1.01 | 0.07 |  | 0.0196 | 0.0004 | 0.0387 | 0.05 |  |
| PECAM1 | 0.99 | 0.84 | 1.17 | 0.92 |  | -0.0008 | -0.0197 | 0.0181 | 0.94 |  |
| NTproBNP | 0.87 | 0.72 | 1.06 | 0.17 |  | 0.0171 | -0.0043 | 0.0385 | 0.12 |  |
| ECP | 0.95 | 0.81 | 1.13 | 0.57 |  | 0.0007 | -0.0182 | 0.0195 | 0.94 |  |
| **Lipids/lipoproteins** |  |  |  |  |  |  |  |  |  |  |
| TC | 0.98 | 0.83 | 1.16 | 0.82 |  | -0.0082 | -0.0275 | 0.0112 | 0.41 |  |
| TG | 0.95 | 0.80 | 1.14 | 0.59 |  | 0.0111 | -0.0081 | 0.0302 | 0.26 |  |
| HDL | 1.15 | 0.97 | 1.36 | 0.10 |  | -0.0230 | -0.0433 | -0.0027 | 0.03 |  |
| LDL | 0.94 | 0.80 | 1.12 | 0.51 |  | -0.0042 | -0.0234 | 0.0150 | 0.67 |  |
| small HDL | 1.02 | 0.87 | 1.20 | 0.78 |  | -0.0024 | -0.0226 | 0.0178 | 0.81 |  |
| large HDL | 1.17 | 1.00 | 1.37 | 0.05 |  | -0.0300 | -0.0513 | -0.0086 | 0.01 |  |
| very small LDL | 1.11 | 0.95 | 1.30 | 0.20 |  | -0.0196 | -0.0401 | 0.0009 | 0.06 |  |
| small LDL | 1.13 | 0.96 | 1.32 | 0.15 |  | -0.0143 | -0.0351 | 0.0066 | 0.18 |  |
| medium LDL | 1.03 | 0.86 | 1.23 | 0.77 |  | -0.0047 | -0.0257 | 0.0163 | 0.66 |  |
| large LDL | 0.85 | 0.70 | 1.02 | 0.08 |  | 0.0081 | -0.0122 | 0.0284 | 0.44 |  |
| small IDL | 0.93 | 0.78 | 1.12 | 0.47 |  | -0.0091 | -0.0295 | 0.0112 | 0.38 |  |
| large IDL | 0.91 | 0.76 | 1.10 | 0.34 |  | -0.0075 | -0.0284 | 0.0134 | 0.48 |  |
| small VLDL | 0.94 | 0.78 | 1.13 | 0.50 |  | -0.0062 | -0.0268 | 0.0143 | 0.55 |  |
| medium VLDL | 0.96 | 0.80 | 1.14 | 0.62 |  | -0.0024 | -0.0227 | 0.0180 | 0.82 |  |
| large VLDL | 0.95 | 0.79 | 1.14 | 0.59 |  | 0.0013 | -0.0191 | 0.0217 | 0.90 |  |
| total VLDL | 0.94 | 0.79 | 1.13 | 0.53 |  | -0.0041 | -0.0245 | 0.0163 | 0.70 |  |
| ^a^ Adjusted for age and sex. Effects were estimated from per one standard deviation of plasma proteins, lipids, and lipoprotein subfractions. Proteins corrected *P*<0.05/88, lipids and lipoprotein subfractions corrected *P*<0.05/16. | | | | | | | | | |  |
|  |  |  |  |  |  |  |  |  |  |  |
| ^b^ Binary logistic regression model (dependent variable: CC [hypolactasia] CT/TT). | | | | | | | | | |  |
| ^c^ Linear regression model (dependent variable: CC, CT, and TT as a continuous variable). | | | | | | | | | |  |

| **Supplementary Table 4.** Associations between the identified plasma proteins, lipids, and lipoprotein subfractions and risks of CVD and CVD mortality ^a^ | | | | | | |  |
| --- | --- | --- | --- | --- | --- | --- | --- |
|  | **Model 1** | | **Model 2** | | **Model 3** | |  |
|  | HR (95% CI) | *P* value | HR (95% CI) | *P* value | HR (95% CI) | *P* value |  |
| **Leptin** |  |  |  |  |  |  |  |
| CHD | 1.09 (0.96, 1.23) | 0.18 | 1.13 (1.00, 1.28) | 0.052 | 1.02 (0.87, 1.19) | 0.80 |  |
| Ischemic stroke | 1.15 (1.00, 1.32) | 0.06 | 1.16 (1.01, 1.33) | 0.04 | 1.18 (0.98, 1.41) | 0.08 |  |
| CVD | 1.10 (1.00, 1.21) | 0.047 | 1.13 (1.03, 1.23) | 0.01 | 1.09 (0.97, 1.23) | 0.15 |  |
| CVD mortality | 1.25 (1.10, 1.42) | <0.001 | 1.29 (1.13, 1.47) | <0.001 | 1.06 (0.90, 1.25) | 0.47 |  |
| **HDL** |  |  |  |  |  |  |  |
| CHD | 0.77 (0.68, 0.87) | <0.0001 | 0.81 (0.71, 0.91) | <0.001 | 0.84 (0.74, 0.95) | <0.01 |  |
| Ischemic stroke | 0.89 (0.78, 1.01) | 0.07 | 0.91 (0.80, 1.03) | 0.14 | 0.92 (0.80, 1.05) | 0.22 |  |
| CVD | 0.81 (0.74, 0.88) | <0.0001 | 0.83 (0.76, 0.91) | <0.0001 | 0.84 (0.77, 0.92) | <0.001 |  |
| CVD mortality | 0.76 (0.67, 0.85) | <0.0001 | 0.80 (0.71, 0.91) | <0.001 | 0.87 (0.76, 0.99) | 0.03 |  |
| **large HDL** |  |  |  |  |  |  |  |
| CHD | 0.78 (0.68, 0.90) | <0.001 | 0.83 (0.72, 0.96) | <0.01 | 0.86 (0.74, 0.99) | 0.04 |  |
| Ischemic stroke | 0.92 (0.80, 1.06) | 0.23 | 0.94 (0.82, 1.09) | 0.40 | 0.95 (0.82, 1.11) | 0.53 |  |
| CVD | 0.81 (0.74, 0.90) | <0.0001 | 0.84 (0.76, 0.94) | <0.01 | 0.86 (0.77, 0.95) | <0.01 |  |
| CVD mortality | 0.75 (0.65, 0.87) | <0.0001 | 0.79 (0.68, 0.92) | <0.01 | 0.86 (0.74, 1.00) | 0.047 |  |
| ^a^ Values are hazard ratios (95% confidence intervals) for per one standard deviation increase unless otherwise indicated. Only included plasma proteins, lipids, and lipoprotein subfractions associated with milk intake after adjusting for lifestyle factors. CVD, cardiovascular disease; CHD, coronary heart disease. | | | | | | |  |
| Model 1: adjusted for age and sex. | | | | | | |  |
| Model 2: model 1 plus leisure-time physical activity, smoking status, alcohol consumption, education, and heredity score (including cancer, myocardial infarction, stroke, and diabetes). | | | | | | |  |
| Model 3: model 2 plus body mass index. | | | | | | |  |
